# Depletion of DNMT1 in differentiated human cells highlights key classes of sensitive genes and an interplay with polycomb repression

**DOI:** 10.1186/s13072-018-0182-4

**Published:** 2018-03-29

**Authors:** Karla M. O’Neill, Rachelle E. Irwin, Sarah-Jayne Mackin, Sara-Jayne Thursby, Avinash Thakur, Ciske Bertens, Laura Masala, Jayne E. P. Loughery, Darragh G. McArt, Colum P. Walsh

**Affiliations:** 1 Genomic Medicine Research Group, Centre for Molecular Biosciences, School of Biomedical Sciences, Ulster University, Cromore Road, Coleraine, BT52 1SA UK; 2 Academie Life Science, Engineering & Design, Saxion University, M.H. Tromplaan 28, 7500 Enschede, Netherlands; 3 Department of Obstetrics and Gynecology, University of Sassari, Via Vienne 2, 7100 Sassari, Italy; 4 Centre for Cancer Research and Cell Biology, Queen’s University Belfast, Belfast, BT9 7AE UK; 5 Present Address: The Wellcome-Wolfson Institute for Experimental Medicine, Queen’s University Belfast, Belfast, BT9 7AE UK; 6 Present Address: Terry Fox Laboratory, BC Cancer Research Centre, 675 West 10th Avenue, Room 13-112, Vancouver, BC V5Z 1L3 Canada

**Keywords:** DNMT1, EZH2, Protocadherin, Body mass, Cancer/testis antigen

## Abstract

**Background:**

DNA methylation plays a vital role in the cell, but loss-of-function mutations of the maintenance methyltransferase *DNMT1* in normal human cells are lethal, precluding target identification, and existing hypomorphic lines are tumour cells. We generated instead a hypomorphic series in normal hTERT-immortalised fibroblasts using stably integrated short hairpin RNA.

**Results:**

Approximately two-thirds of sites showed demethylation as expected, with one-third showing hypermethylation, and targets were shared between the three independently derived lines. Enrichment analysis indicated significant losses at promoters and gene bodies with four gene classes most affected: (1) protocadherins, which are key to neural cell identity; (2) genes involved in fat homoeostasis/body mass determination; (3) olfactory receptors and (4) cancer/testis antigen (CTA) genes. Overall effects on transcription were relatively small in these fibroblasts, but CTA genes showed robust derepression. Comparison with siRNA-treated cells indicated that shRNA lines show substantial remethylation over time. Regions showing persistent hypomethylation in the shRNA lines were associated with polycomb repression and were derepressed on addition of an EZH2 inhibitor. Persistent hypermethylation in shRNA lines was, in contrast, associated with poised promoters.

**Conclusions:**

We have assessed for the first time the effects of chronic depletion of DNMT1 in an untransformed, differentiated human cell type. Our results suggest polycomb marking blocks remethylation and indicate the sensitivity of key neural, adipose and cancer-associated genes to loss of maintenance methylation activity.

**Electronic supplementary material:**

The online version of this article (10.1186/s13072-018-0182-4) contains supplementary material, which is available to authorized users.

## Background

DNA methylation is an important mechanism for epigenetic regulation of genes in both mouse and human [1]. It occurs mainly at the CpG dinucleotide, and methylation at this symmetrical site is efficiently maintained during replication by the action of the DNA methyltransferase 1 (DNMT1) enzyme [2]. Methylation is known to play an important role in regulating imprinted loci [3], genes on the inactive X chromosome [4] and germline-specific genes [5] in mouse.

Where methylation occurs at the promoter of a gene, it is strongly associated with the silencing of transcription, particularly if there is a high density of CpGs, a so-called CpG island (CGI). However, studies have shown that most CGI are intrinsically protected from methylation [6, 7] and only a small number shows dynamic changes during development, mostly in the three classes mentioned above [5, 8], though there may be others which have not yet been clearly defined. As you move outward from an island, the shores and shelves show higher levels of methylation and greater dynamic response [9], though here the link to changes in gene activity is less clear [10]. Methylation is also associated with larger regions of inert chromatin, such as the inactive X, pericentromeric repeats and regions rich in transposable elements [1], generally consistent with a repressive role. Recent genome-wide surveys have also indicated that high levels of methylation are found in the bodies of active genes, where they may facilitate transcription [11, 12]. In keeping with this, we and others recently showed that artificially decreasing intragenic methylation levels reduced steady-state transcript levels, consistent with a positive role for methylation in the gene body [11–13].

Another major system for epigenetic repression is via histone modification, particularly by the polycomb group of proteins, with EZH2 being one of the main enzymes involved [14]. A number of studies suggest an interplay between polycomb- and DNMT-mediated repression, with a generally negative correlation between DNA methylation and the H3K27me3 mark deposited by EZH2 [15, 16]. Supporting this, a loss of DNA methylation caused a reshaping of the histone landscape and derepression of some polycomb targets in mouse ES cells [17], suggesting that DNA methylation helps to determine where polycomb marks are deposited.

While DNMT1 is the main maintenance methyltransferase, there also appears to be an important role for the de novo enzymes DNMT3A and DNMT3B in complementing that activity at some loci [18, 19]. In order to clarify which genes are most sensitive to DNMT1 loss in human, a number of studies have been carried out using mutations within the gene to assess the effects of loss of methylation [19–22]. While this has been a fruitful approach in mouse embryonic stem (ES) cells, where null mutants are tolerated, differentiation of the mouse cells leads to cell death [20, 22, 23], whereas DNMT1 disruption in human ES cells is not tolerated even in undifferentiated cells [24]. Genetic ablation in adult differentiated cells also leads to cell death within a few cell cycles, before passive demethylation of the genome can occur [23, 25]. One of the best-studied systems in humans consists of HCT116 colon cancer cells carrying a hypomorphic allele in the DNMT1 gene together with a DNMT3B knockout (HCT116 DKO cells) [26–28]. Blattler et al. [29] found that there was widespread and relatively uniform demethylation across the genome in the DKO cells, with small effects at CGI (most of which are normally unmethylated anyway) and relatively few genes showing derepression. There was no enrichment by gene ontology (GO) analysis, but some effect at enhancers: however, this is complicated by the presence of the DNMT3B knockout alleles. Acute depletion of DNMT1 using an siRNA-mediated approach in embryonal carcinoma cells also found regions of low CpG density (open sea, shelf) to be the most affected by loss of methylation [70]. Among the small number of dysregulated genes, there was some enrichment for cell morphogenesis and phosphorylation pathways.

Neither of these cancer cell lines, however, are a good model for the normal differentiated cell as they are transformed, aneuploid, hypermethylated, and contain a number of different mutations in key regulatory genes. Additionally, acute depletion of DNMT1 results in cell cycle delay, triggering of the DNA damage response and increased rates of cell death [24, 25, 30], making it difficult to separate acute and chronic effects.

To circumvent some of the difficulties outlined above, we generated a series of isogenic human cell lines derived from the hTERT-immortalised normal fibroblast line hTERT1604 as previously described [30]. These are normosomic and non-transformed, and by using a stably incorporated plasmid with an shRNA targeting *DNMT1* we were able to isolate a number of clonally derived lines to allow identification of any cell line-specific effects. While these showed initially the range of shared features indicative of a global response to the loss of this critical regulator, including cell cycle delay, demethylation of imprinted genes and others, they could be cultured for longer under selection [30], allowing identification of loci with particular sensitivity for decreased maintenance methyltransferase activity. Here we set out to completely characterise the methylation changes seen in the cell lines using the Illumina Infinium HumanMethylation450 BeadChip (450k) array platform [31] and subsequent analysis using the RnBeads pipeline [32]. These approaches were chosen due to their high reproducibility and low inter-operator variability, ensuring the reliable and sensitive detection of alterations in methylation. A sample of the observations was then further verified using locus-specific assays. In addition and for the same reasons, we used the HT-12 Expression v4 BeadChip array, to assay changes in transcription in our cell lines.

## Methods

### Cell culture

The parental or wild-type (WT) adherent hTERT1604 lung fibroblast cell line [33] was cultured in 4.5 g/l glucose DMEM (Thermofisher, Loughborough, UK) supplemented with 10% FBS and 2× NEAA (Gibco/Thermofisher). Generation of the hTERT1604 cell lines stably depleted of DNMT1 using a pSilencer construct (Thermofisher) has been previously described [30]. Knockdown (KD) cells were maintained as for WT, but medium was supplemented with 150 μg/ml hygromycin B (Invitrogen/Thermofisher, Paisley, UK), which was removed at least 48 h before any experimental procedure. Treatment of cells with siRNA for 24 h was as previously described [34]: for the pulse-chase experiment cells were afterwards allowed to recover in normal media and passaged as required for up to 36 days. The siRNA (Dharmacon ON-TARGETplus SMARTpool) for *DNMT1* and *DNMT3B*, as well as scrambled control, was obtained from Invitrogen/Thermofisher. HCT116 and double knockout (DKO) cells [27] were cultured in 1 g/l glucose DMEM (Gibco) supplemented with 10% FBS and 1× NEAA (Gibco). DZNeP (Sigma-Aldrich, Dorset, UK) was used at a final concentration of 1 μM.

### DNA extraction and bisulphite conversion

Genomic DNA was harvested from cells in log phase of growth. Samples were incubated overnight at 55 °C in lysis buffer [50 mM Tris pH 8, 0.1 M EDTA (both Sigma-Aldrich), 0.5% SDS, 0.2 mg/ml proteinase K (Roche, West Sussex, UK)], with rotation, and DNA was subsequently isolated using the standard phenol/chloroform/isoamyl alcohol (25:24:1 pH8, Sigma-Aldrich) extraction method. DNA quality was verified using gel electrophoresis and UV absorbance measurements at 260/280 and 260/230 nm using a Nanodrop UV spectrophotometer (Labtech International, Ringmer, UK). Bisulphite conversion of 500 ng of DNA was carried out using the EpiTect bisulphite kit (Qiagen, Crawley, UK) according to the manufacturer’s instructions.

### Hybridisation to 450K array and bioinformatic analyses

Three samples from each cell line were used to prepare DNA, with at least one biological repeat in each set. DNA was assessed for purity and integrity as above prior to quantification using the Quant-iT PicoGreen dsDNA assay kit (Thermo Fisher Scientific) as per manufacturer’s instructions. In total, 500 ng of high-quality bisulphite-converted (Zymo Research) DNA was checked for purity and fragmentation on a bioanalyser and then loaded on the Infinium HumanMethylation450 BeadChip [31] and imaged using an Illumina iScan (Cambridge Genomic Services). Output files in IDAT format were processed using the RnBeads [32] methylation analysis package (v1.0.0) which carries out all the analysis from import to differential methylation within the R platform (3.2.0). Briefly, quality control used the built-in probes on the array and included filtering out of probes containing SNPs, and checking for hybridisation performance. Normalisation was then carried out using the SWAN method in minfi [35] after background subtraction with methylumi.noob. The exploratory analysis module was used to generate probe density distributions and scatter graphs. The differential methylation analyses was based on a combined ranking score, which combined absolute effect size, relative effect sizes and p-values from statistical modelling into one score where rank is computed as the most conservative value among mean difference in means, mean in quotients and combined *p* value across sites in the region: the enrichment analysis used the combined rank among the 1000 best-ranking regions and a hypergeometric test to identify GO terms in the AmiGO 2 database [36]. Pairwise comparison of triplicate samples from each cell line against WT hTERT was also made to determine change in beta value and associated combined p-value, adjusted for multiple comparison using false discovery rate (FDR). Some tailored analyses were also carried out using custom scripts in R. Additional GO studies were performed using DAVID (v6.7) [37].

We used the GALAXY platform [38] to map sites showing highly reproducible changes (FDR < 0.05) against the locations of RefSeq genes or ChromHMM regions on the UCSC genome browser [39] for each cell line. GO category genes which showed changes in methylation at multiple sites in more than one KD cell line were scored as true hits (Yes in the FDR column), while GO categories with few or no sites reproducibly altered across replicates (FDR > 0.05) or where methylation changes were small (< 0.1 *β*), inconsistent in direction, or not found in more than one KD cell line, were scored as false positives. Absolute *β* levels were used to measure median methylation across genes of interest using custom workflows in GALAXY, with further statistical analyses in Statistical Package for the Social Sciences software (SPSS) version 22.0 (SPSS UK Ltd).

### Locus-specific methylation analysis

Amplification was carried out using the PyroMark PCR kit (Qiagen) with 2 μl bisulphite-converted DNA, 12.5 μl MasterMix, 2.5 μl CoralLoad Concentrate, 1.25 μl each primer (10 μM) and 5.5 μl nuclease-free H_2_o using the following conditions: 15 min at 95 °C followed by 45 cycles of 94 °C for 30 s, 56 °C for 30 s, 72 °C for 30 s and a final elongation step of 72 °C for 10 min. Pyrosequencing was carried out on the PyroMark Q24 System, according to the manufacturer’s instructions (Qiagen). Most assays were designed in-house using the PyroMark Assay Design software 2.0 (*LEP*, *MAGEA12*, *OR10J5*, *OR51E2*, *OR2AG1*, *PCDHA2*, *PCDHC4*, *UGT1A1*, *UGT1A4*) prior to synthesis (Metabion, Germany): see Additional file 1: Table S1 for details: *DAZL*, *SYCP3*, *D4Z4* and *NBL2* were as described [34, 40]. In some cases, pre-designed pyrosequencing primers were obtained from Qiagen (*GABRQ* PM00133483, *GHSR* PM00014350, *SNRPN* PM00168252). Clonal analysis was carried out as previously described [30].

### Hybridisation to HT-12 microarray and bioinformatic analyses

Total RNA was extracted using the RNeasy minikit (Qiagen) as per manufacturer’s instructions, including a DNase step. RNA integrity was verified via gel electrophoresis, and quality and quantity were verified using a SpectroStar (BMG Labtech, Aylesbury, UK) and a bioanalyser (Agilent Technologies, Cheadle, UK). Two hundred nanograms of total RNA underwent linear amplification using the Illumina TotalPrep RNA Amplification Kit (Life Technologies/Thermofisher, Paisley, UK) following the manufacturer’s instructions. Microarray experiments were performed at Cambridge Genomic Services, University of Cambridge, using the HumanHT-12 v4 Expression BeadChip (Illumina, Chesterford, UK). After scanning the data were loaded in GenomeStudio (Illumina) and then processed in R (version 3.2.2). The data were filtered to remove any non-expressed probes using the detection p-value from Illumina, transformed using the variance stabilization transformation (VST) from lumi and normalised using the quantile method. Comparisons were made using the limma package with results corrected for multiple testing using false discovery rate (FDR) testing.

### RNA and protein analysis

Transcriptional assays at individual loci using RT- and RT-qPCR were carried out essentially as in [34]: primer sequences are listed in Additional file 1: Table S1. Protein was extracted from cells growing in log phase using protein extraction buffer (50 mM Tris–HCl, 150 mM NaCl, 1% Triton-X, 10% glycerol, 5 mM EDTA; all Sigma-Aldrich) and 0.5 µl protease inhibitor mix (Sigma-Aldrich). For Western blotting, 30 μg protein was denatured in the presence of 5 μl 4× LDS sample buffer (Invitrogen) and 2 μl 10× reducing agent (Invitrogen) in a total volume of 20 μl nuclease-free water (Qiagen) via incubation at 70 °C. Proteins were separated by SDS-PAGE and then electroblotted onto a nitrocellulose membrane (Invitrogen) and blocked in 5% non-fat milk for 1 h at room temperature (RT). Membranes were incubated with anti-DNMT1 (a kind gift from Guoliang Xu) and anti-*β*-actin (Abcam ab8226) overnight at 4 °C, followed by HRP-conjugated secondary antibody incubation at RT using ECL (Invitrogen).

### Statistical analysis

Statistical analysis was performed by the RnBeads package, or separately in Excel (Microsoft Office Professional Plus 2013), Prism (Graphpad) or SPSS (v22.0). Experiments were carried out in triplicate and included at least one biological replicate. PCR results were analysed using Student’s paired *t*-test. Pyrosequencing results were analysed by ANOVA within representative runs and using Student’s *t*-test on the average of multiple runs. Error bars on all graphs show standard error of the mean (SEM) or in the case of HT12 array data, 95% confidence interval (CI), unless otherwise stated. Asterisks are used to represent probability scores as follows: *p < 0.05; **p < 0.01; ***p < 0.005 or n.s. not significant.

## Results

### Generation of isogenic hTERT1604 fibroblast cell lines

**Fig. 1:**
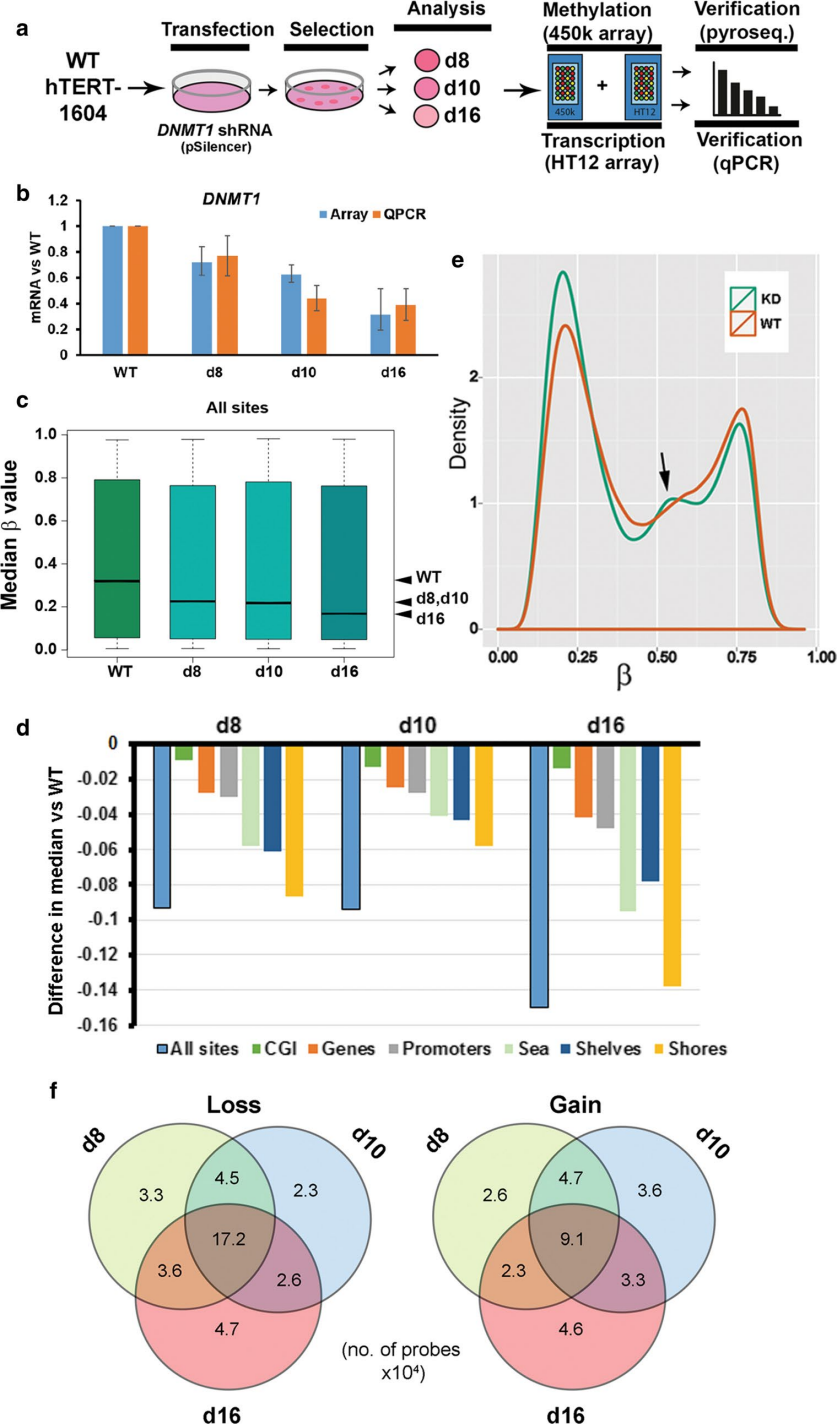
Cell line generation and overall changes seen in methylation levels. **a** Experimental approach: WT hTERT1604 fibroblasts were transfected with shRNA-containing plasmid and grown in selective medium; colonies of resistant cells were expanded, and three (d8, d10, d16) showing reduced DNMT1 levels were then analysed using genome-wide methylation and transcription arrays on the Illumina platform. **b** Levels of *DNMT1* mRNA in cell lines from array and qPCR: error bars represent 95% confidence intervals around median, and standard error of the mean (SEM), respectively. All three knockdown (KD) lines were significantly depleted at *p* < 0.05 for both assays (except d8 array). **c** Overall methylation levels in WT and KD cells as measured by 450K: a *β* value of 1 equates to 100% methylation. Median values are indicated by the line, and whiskers represent interquartile range. The positions of the medians are also indicated at right (arrowheads). **d** The difference in median *β* value between each KD cell line and WT is shown first for all sites assayed (see **c** above) and then for each type of genomic element. CGI, CpG island; shore, region adjacent to CGI; shelf, adjacent to shore; sea, all other. See also Additional file 3: Fig. S2B. **e** Probe density distributions; in KD there is a decrease in the number of fully methylated sites (*β* closer to 1) and an increase in the number of unmethylated sites (*β* closer to 0), as well as in probes showing intermediate levels of methylation (arrow). **f** Numbers of sites (×10^4^) showing significant changes in methylation (FDR < 0.05) compared to WT: the set of common sites is largest in each case, with close to twice as many sites commonly losing methylation in comparison with those gaining

Isogenic lines carrying an shRNA construct targeting *DNMT1* were generated by transfecting the hTERT-immortalised human lung fibroblast cell line hTERT-1604 with pSilencer plasmid containing an shRNA (Fig. 1a). The generation and initial characterisation of these isogenic cell lines have been previously described [30]. Here we took two sublines typical of the intermediate levels of knockdown (KD) seen (d8 and d10) as well as one line (d16) with relatively low levels of mRNA, with good agreement between reverse transcription quantitative PCR (qPCR) and array results (Fig. 1b; all *p *< 0.05 except d8 array). We also confirmed knockdown at the protein level using Western blotting, with HCT116 cells mutated in DNMT1 and DNMT3B [27] as controls (Additional file 3: Fig. S2A).

### Characterisation of overall changes in absolute methylation levels in depleted lines

Using the 450K array [31] and processing in RnBeads [32] to assess methylation levels across the genome (Fig. 1c), there was still a wide range of methylation values (given for the array as a value β ranging from 0 to 1) in KD lines as compared to WT, but the median values were decreased as expected in all three with d8 being comparable to d10, while d16 was lower (arrowheads at right). Principle components analysis and examination of the sites showing greatest differences in methylation between the stable lines confirmed that d8 and d10 were most similar (Additional file 2: Fig. S1). Probes on the array were annotated by location relative to genomic features, and while all regions showed a decrease in methylation, the difference in median values was smallest for CGI, which were unmethylated anyway in parental cells (*β* < 0.1 in WT), while the separation in medians was greatest at shelves and shores, where methylation levels were higher (Additional file 3: Fig. S2B). This can most clearly be seen by plotting the difference in medians (Fig. 1d). Both WT and the KD cell lines showed the typical bimodal probe density distribution pattern reported in most cell types [31] (Fig. 1e). Overall, there was an increase in the numbers of less methylated probes (*β* < 0.25) in the KD cell lines and a decrease in the numbers of highly methylated probes (*β* > 0.65). For individual regions CGI again showed the smallest change, while gene bodies (genes) appeared most altered (Additional file 3: Fig. S2C).

To determine whether methylation was lost stochastically in each KD cell line given the variation seen (Additional file 2: Fig. S1), or was more targeted, we determined the degree to which affected sites were shared between the three cell lines (Fig. 1f). The largest set of sites losing methylation (17.2 × 10^4^) was that shared between all three KD lines, supporting a non-random loss. A spike in numbers of probes showing intermediate levels of methylation (*β* ~ 0.50) in KD cell lines in the density profile plot (Fig. 1e, arrow) had indicated that a possible gain in methylation might also be occurring at some sites. Analysis showed that a substantial number (9.1 × 10^4^) of sites gaining methylation are shared between all three KD lines, indicating reproducible gains in methylation at particular CpGs.

### Overall pattern of sites showing significant differential methylation on DNMT1 depletion

We compared WT cells to all three KD lines using the RnBeads package in R and combined rank scoring (see methods). This confirmed that d16 has the greatest number of demethylated sites using a false discovery rate (FDR) cut-off of *p* < 0.05, but at *p* < 0.001 all three lines have comparable numbers of hypo- and hypermethylated sites (Additional file 4: Fig. S3A), with more sites losing than gaining. An analysis of the 1000 best-ranking sites highlights sites common to all three KD lines (Additional file 4: Fig. S3B), confirming that there are large numbers of sites which respond in the same way in each KD, with an excess of probes showing loss over gain.

We then looked to see whether shared probes were enriched in any particular gene region. As we were interested in changes which might cause altered transcription, we focussed on CGI, promoters and gene bodies (hereafter genes) rather than shores, shelves or open sea, where correlations with transcriptional output are harder to assess. Using a hypergeometric test in RnBeads, both promoters and genes, but not CGI, showed significant enrichment in demethylated probes for particular gene ontology (GO) terms. Table 1 indicates the top 3 ontology classes under biological process (BP) and molecular function (MF). For loss of methylation, examining common genes and processes suggested that three classes of genes were common to the enriched GO terms, which we grouped as follows: (1) genes involved in neuroepithelial differentiation; (2) genes involved in fat homoeostasis/body mass (FBM); and (3) olfactory receptor genes (groups 1–3 in Table 1), all of which will be dealt with below. The only orphan GO term whose members had multiple high-confidence demethylated sites was GO:0007506 gonadal mesoderm formation, which largely consists of members of the *TSPY* gene family on the Y chromosome. For gain of methylation, the same was true in that a relatively small number of histone modifier genes (group 4), represented under several GO terms, were responsible for many of the hits. In addition, the GO terms for glucuronosyltransferase activity (GO:0015020) and for regulation of megakaryocyte differentiation were also represented (Table 1). These were then curated by looking for sites showing reproducible changes (FDR < 0.05) in all KD lines (described more fully in “Methods” section), which indicated strong support [Yes (Y) in confirm column, Table 1] for all GO categories showing loss, but only in two showing gain (GO:0015020 and GO:0004984). We then set about verifying these targets.

**Table 1:** Gene ontology analysis for differentially methylated sites

Type	GO FID	*P*	OR	Ex	Obs	Total	GO Term	Grp	confirm
*Loss*									
Promoter									
BP	0098609	0.0011	3.0454	4.2148	12	189	Cell–cell adhesion	1	Y
	0007156	0.0011	3.4722	3.0998	10	139	Homophilic cell adhesion via plasma membrane	1	Y
	0010982	0.0015	88.2036	0.0669	2	3	Regulation of high-density lipoprotein particle clearance	2	Y
MF	0004888	0.0001	1.9709	24.2681	44	1055	Transmembrane signalling receptor activity	3	Y
	0005509	0.0001	2.2488	14.3768	30	625	Calcium ion binding	1	Y
	0004871	0.0003	1.7441	33.6302	54	1462	Signal transducer activity	3	Y
Gene									
BP	0007506	0	130.3775	0.1339	5	7	*Gonadal mesoderm development*		Y
	0032375	0.0001	25.9783	0.2295	4	12	Negative regulation of cholesterol transport	2	Y
	0045409	0.0001	77.705	0.0956	3	5	Negative regulation of interleukin-6 biosynthetic process	2	Y
MF	0008083	0.0009	3.5742	3.0015	10	158	Growth factor activity	3	Y
	0004984	0.0014	2.5939	6.136	15	323	Olfactory receptor activity	3	Y
	0038023	0.0014	1.7776	22.9102	38	1206	Signalling receptor activity	3	Y
*Gain*									
Promoter									
BP	0035574	0	443.1106	0.4729	14	15	Histone H4-K20 demethylation	4	N
	0045653	0	147.6833	0.5359	14	17	Negative regulation of megakaryocyte differentiation		N
	0016577	0	26.4022	1.0404	15	33	Histone demethylation	4	N
MF	0035575	0	452.3692	0.4637	14	15	Histone demethylase activity (H4-K20 specific)	4	N
	0032451	0	21.0879	1.1747	15	38	Demethylase activity	4	N
	0015020	0	10.1109	0.8965	7	29	*Glucuronosyltransferase activity*		Y
Gene									
BP	0035574	0	280.0725	0.4039	14	16	Histone H4-K20 demethylation	4	N
	0045653	0	140.0181	0.4544	14	18	Negative regulation of megakaryocyte differentiation		N
	0006335	0	31.0869	0.8078	14	32	DNA replication-dependent nucleosome assembly	4	N
MF	0035575	0	287.2955	0.3942	14	16	Histone demethylase activity (H4-K20 specific)	4	N
	0032451	0	24.654	0.9856	15	40	Demethylase activity	4	N
	0004984	0	4.4768	7.9586	31	323	Olfactory receptor activity	3	Y

*BP* biological process, *MF* molecular function, *GO FID* gene ontology family identification code, *P* probability value, *OR* odds ratio, *Ex* expected number of hits, *Obs* observed number, *Total* total number of genes in that family, *Grp*-see below; confirm Y/N, confirmation given by FDR tracks Yes/No

Groups (Grp): 1 = neuroepithelium; 2 = Fat homoeostasis/body mass (FBM); 3 = olfactory receptor; 4 = histone modifier

### Loss of methylation at the protocadherin gamma gene cluster particularly affects the A and B class variable genes

**Fig. 2:**
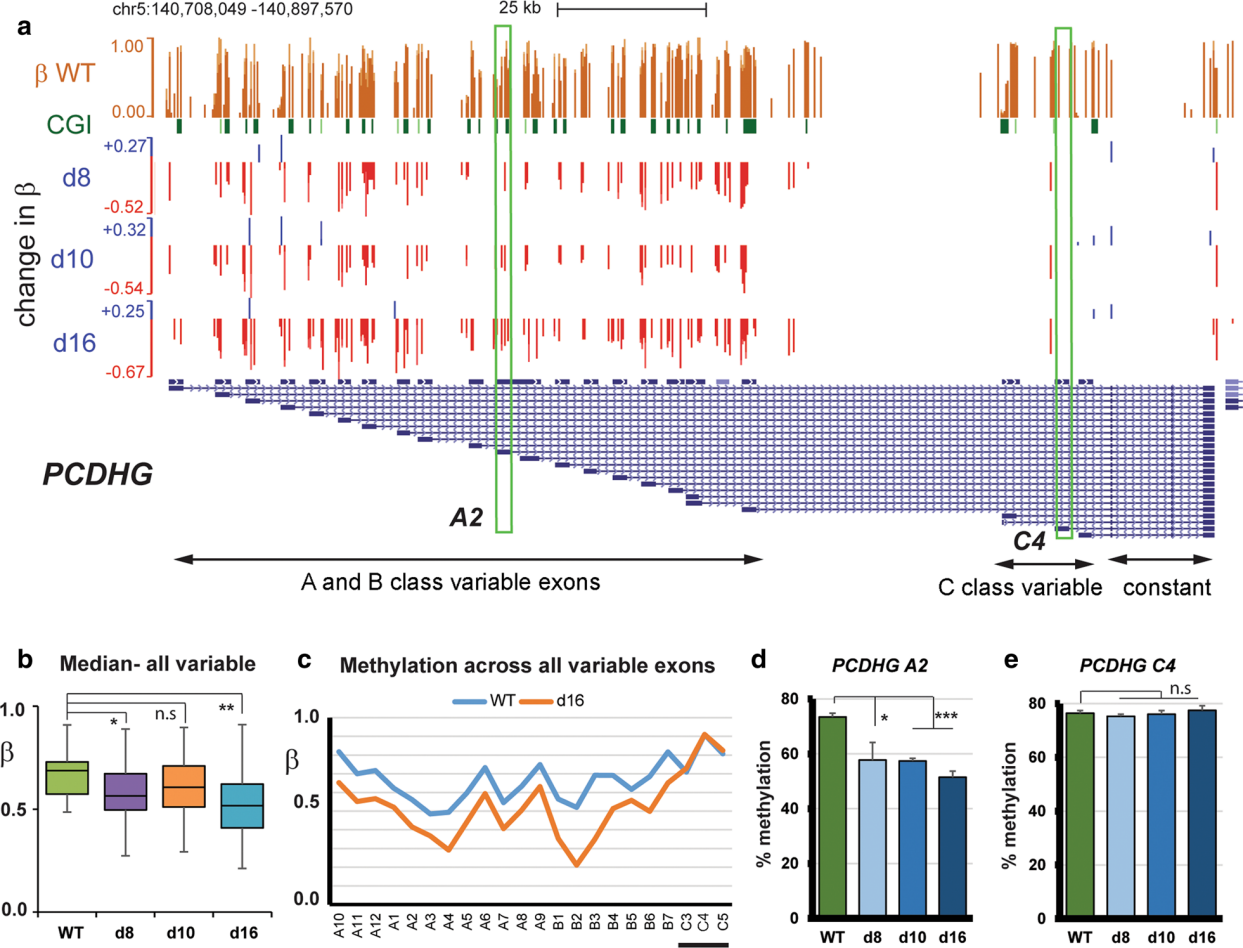
Loss of methylation at the protocadherin *γ* (*PCDHG*) cluster of neuroepithelial identity genes. **a** Structure of the *PCDHG* cluster showing the 5′ variable exons (A, B and C classes) which are spliced to the 3′ constant exons (right). The top track (amber) shows absolute *β* values in the WT fibroblast cells from the 450K array, which range from 1(fully methylated) to zero (unmethylated). Only the sites showing significant differences from WT (FDR < 0.05) in each cell line are shown in the three tracks below, with decreases in red representing loss of methylation, and gains in blue. The size of the bar is proportional to the magnitude of change: maxima and minima are indicated on the scales at left. The locations of CpG islands (CGI) are also shown. Pyroassay locations are boxed. **b** Median *β* values for all variable exons. Significant differences (Mann–Whitney *U*) are indicated: *p < 0.05; **p < 0.1; n.s., not significant. **c** Methylation at each exon in WT and d16 cells obtained by taking the median of the absolute *β* value for all probes at that exon. The variable class C exons are underlined. **d** Average methylation values in WT and KD cells obtained from a pyrosequencing assay (pyroassay) designed to cover CpGs in the A2 exon. Bars represent SEM; ***p < 0.001, *t*-test. **e** Methylation at the C4 variable exon by pyroassay, shown as a control

A main contributor to the enrichment of neuroepithelial genes are the protocadherin genes. Protocadherin *α*, *β* and *γ* (*PCDHA*, *PCDHB* and *PCDHG*) genes are located in three linked clusters on chromosome 5 and give rise to neural cell–cell adhesion proteins, with significant loss of methylation across the whole region in all three cell lines (Additional file 4: Fig. S3C). The *α* and *γ* proteins have a variable extra-cellular recognition domain, either A, B or C-type, attached to a constant transmembrane and intracellular domain. This is achieved at the gene level by alternative 5′ exons encoding the variable region being spliced to the constant region exons. Figure 2a shows the tracks containing sites with significant (FDR < 0.05) methylation differences between KD and WT cells for the *PCDHG* cluster. These reveal loss of methylation (in red in Fig. 2a) at most A and B class variable exons in all three KD cell lines, but not at the C class variable or the constant exons. Array probes were present in this region, and examination of the absolute rather than relative methylation (amber, top track in Fig. 2a) confirmed high levels of methylation in WT, where median β values were high for all variable exons (Fig. 2b). Methylation decreased in all three KD lines, with d10 showing the least effect (Fig. 2b). Methylation was substantially altered at all A and B class variable exons, but not at the C class (Fig. 2c). We could experimentally verify the loss of methylation at A2 (Fig. 2d), and no change at C4 (Fig. 2e), using pyrosequencing assays (pyroassay).

Some demethylation of other neuroepithelial genes in this GO category was also seen from the array, such as *S100P*, *ROBO1* and *PAX6*, with significant (*p* < 0.05) demethylation of *S100P* in two-thirds of KD cell lines confirmed by pyrosequencing (not shown).

### Loss of methylation at other targets including fat homoeostasis/body mass (FBM) genes

**Fig. 3:**
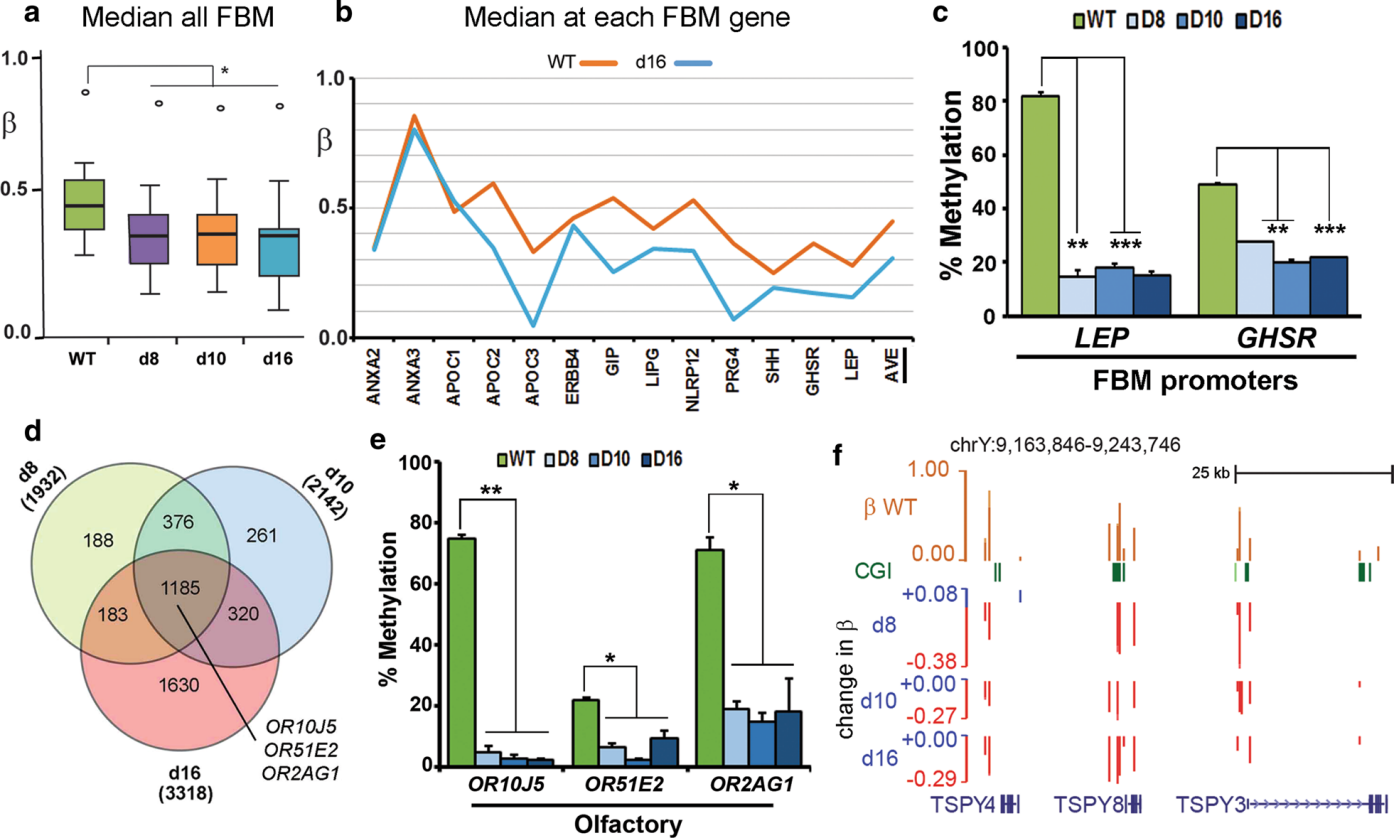
Loss of methylation at fat homoeostasis/body mass (FBM) genes, olfactory receptors and the TSPY genes. **a** Median *β* values for all FBM genes (following curation) in WT and KD cell lines; significant differences (Mann–Whitney *U*) are indicated. **b** Median methylation values at each FBM gene in WT and d16 cells. **c** Average methylation levels obtained from pyroassays at the Leptin (*LEP*) and Ghrelin/growth factor receptor secretagogue (*GSHR*) promoters. **d** CpG located in promoters which showed highly reproducible loss of methylation (FDR < 0.05) were identified. The set of sites common to all three KD cell lines (*n* = 1185) was found to be enriched for olfactory receptors (such as the three indicated) using the DAVID tool. **e** Pyroassays designed for the three olfactory receptor genes from (**d**) confirmed methylation was consistently reduced across all KD cell lines. **f** Browser view showing loss of methylation (red) at the CGI-containing promoters for members of the *TSPY* family on the Y chromosome

Another class of genes showing enrichment all appear to be involved in some aspect of triglyceride processing, energy homoeostasis and body weight regulation (Table 1), including leptin (*LEP*), ghrelin/growth hormone secretagogue receptor *(GHSR)* and genes encoding the very low density lipoproteins *APOC1*, *APOC2* and *APOC3*. Median levels of methylation in the gene bodies were approximately 45% in WT (*β* = 0.45) and showed significant (*p* < 0.05) decreases in the KD lines (Fig. 3a). Most individual genes also showed substantial loss, with the exception of the *ANXA* genes (Fig. 3b). Loss of methylation at the *LEP* and *GHSR* promoters was confirmed using pyroassay (Fig. 3c).

Olfactory receptor (OR) genes appeared in a number of GO categories as having lost methylation, though some gains in the gene body were also indicated (Table 1). ORs encode G protein-coupled receptor proteins and are members of a large gene family, many of which are grouped into major clusters, particularly on chromosome 11 [41]. To buffer against stochastic effects due to the large gene family involved, we carried out a second analysis starting instead with sites in promoters showing reliable methylation loss compared to WT (FDR < 0.05) in the triplicates of each KD line and then overlapping these (Fig. 3d) to see which sites were common to all three KD cell lines (Additional file 5: Table S2). Ontology analysis of these common sites using DAVID independently highlighted signalling receptor genes and more particularly olfactory receptors (*n* = 21). This group of OR genes also showed significant demethylation compared to WT (Kruskal–Wallis, *p *< 0.05) across the genes when median methylation at all available probes was analysed (Additional file 4: Fig. S3D). We chose three of these genes—*OR10J5*, *OR51E2* and *OR2AG1*—located on different chromosomes and could verify loss of methylation in all KD lines (Fig. 3e).

The final GO category of genes (GOFMID:0007506) showing loss of methylation (Table 1) consists largely of the *TSPY* gene family (*TSPY1*-*4*, *8* and *10*) located on the Y chromosome and thought to be implicated in both normal gonadal development and in gonadoblastoma [42]. These also showed clear evidence of demethylation (Fig. 3f).

### Gains in methylation affect the UGT1A locus

**Fig. 4:**
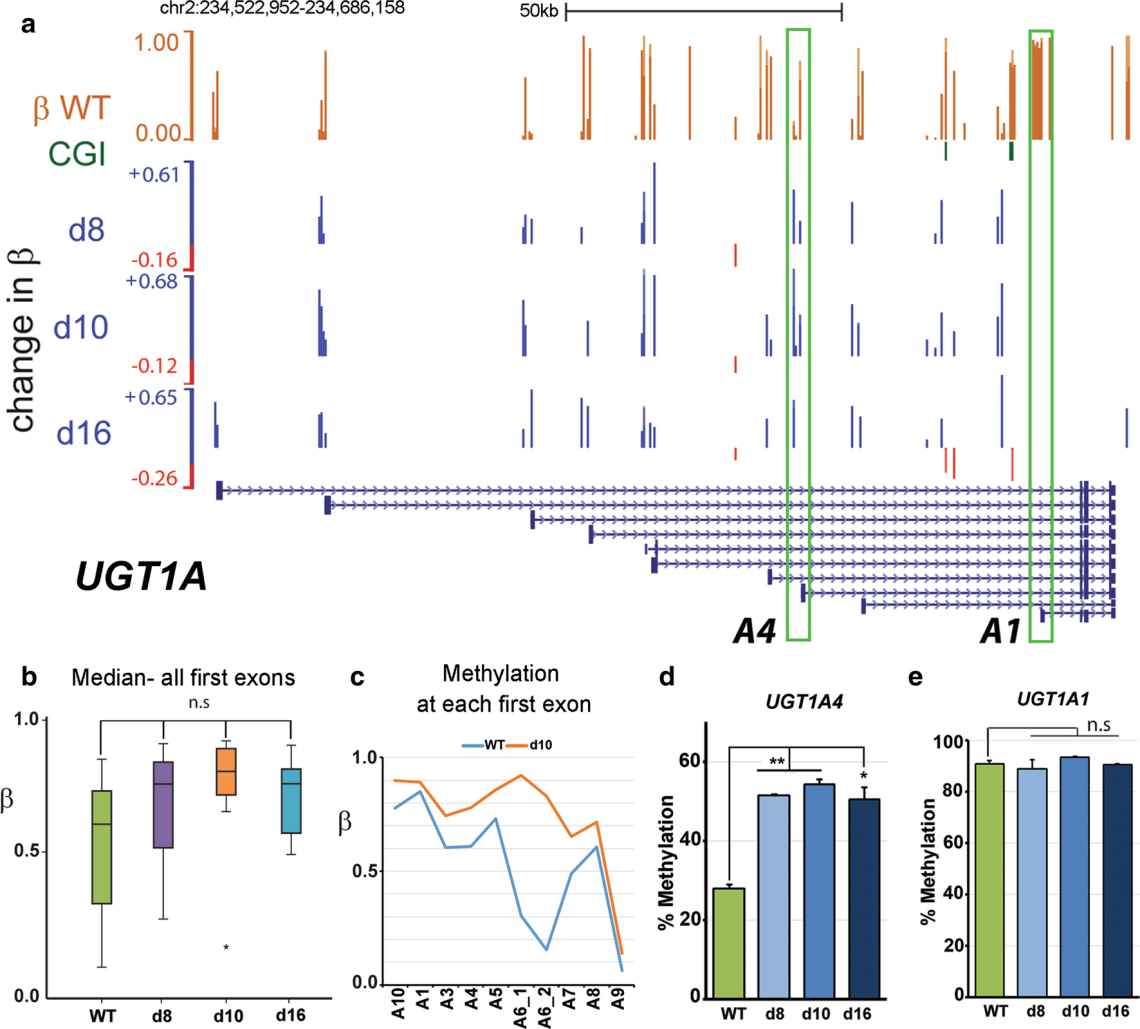
Gains in methylation at the clustered *UGT1A* locus. **a** Structure of the *UGT1A* cluster showing the 5′ variable exons (*UGT1A1–UGT1A10*) which are spliced to the 3′ exons (right). Key to tracks as before; pyroassay locations (*UGT1A1* and *UGT1A4*) are boxed. **b** Median *β* values for all first exons: though medians are higher in KD lines these failed to reach statistical significance. **c** Median absolute *β* values at individual first exons in WT and d16 cells. **d** Average methylation values in WT and KD cells obtained from a pyrosequencing assay (pyroassay) designed to cover CpGs in the A4 exon. **e** Methylation by pyroassay at the A1 exon, shown as a control

As indicated above, with respect to gains in methylation only two of the GO classes identified in the genome-wide screen (Table 1) contained multiple sites showing significant gains in methylation (FDR < 0.05, > 0.1 gain in *β*). One of these was the olfactory genes, discussed above: the other GO term GO:0015020 was largely comprised of members of the *UGT1A* family. This gene family has a similar structure to the *PCDHG* cluster, where unique alternate 5′ exons splice to common 3′ exons, but in this case codes for a series of nine UDP-glucuronosyltransferase enzymes (UGTs). Substantial gains in methylation can be seen at the upstream promoters controlling the 5′ exons (Fig. 4a), most of which lack CGI. Median methylation levels also showed clear increases overall in the KD lines (Fig. 4b), though these did not reach significance. Most individual exons also showed a sharp increase (Fig. 4c), with A1 being a clear exception in all lines. We confirmed a significant gain in methylation in each cell line at A4 (Fig. 4d) but no alteration at A1 (Fig. 4e). In contrast to the clear gains in all three lines for *UGT1A*, the histone modifier group also identified as gaining methylation (Table 1, group 4) contained few FDR-supported sites and these often did not overlap between cell lines, with median *β* levels also not differing significantly (Additional file 4: Fig. S3E).

### A cluster of loci showing gain of methylation on the X chromosome

**Fig. 5:**
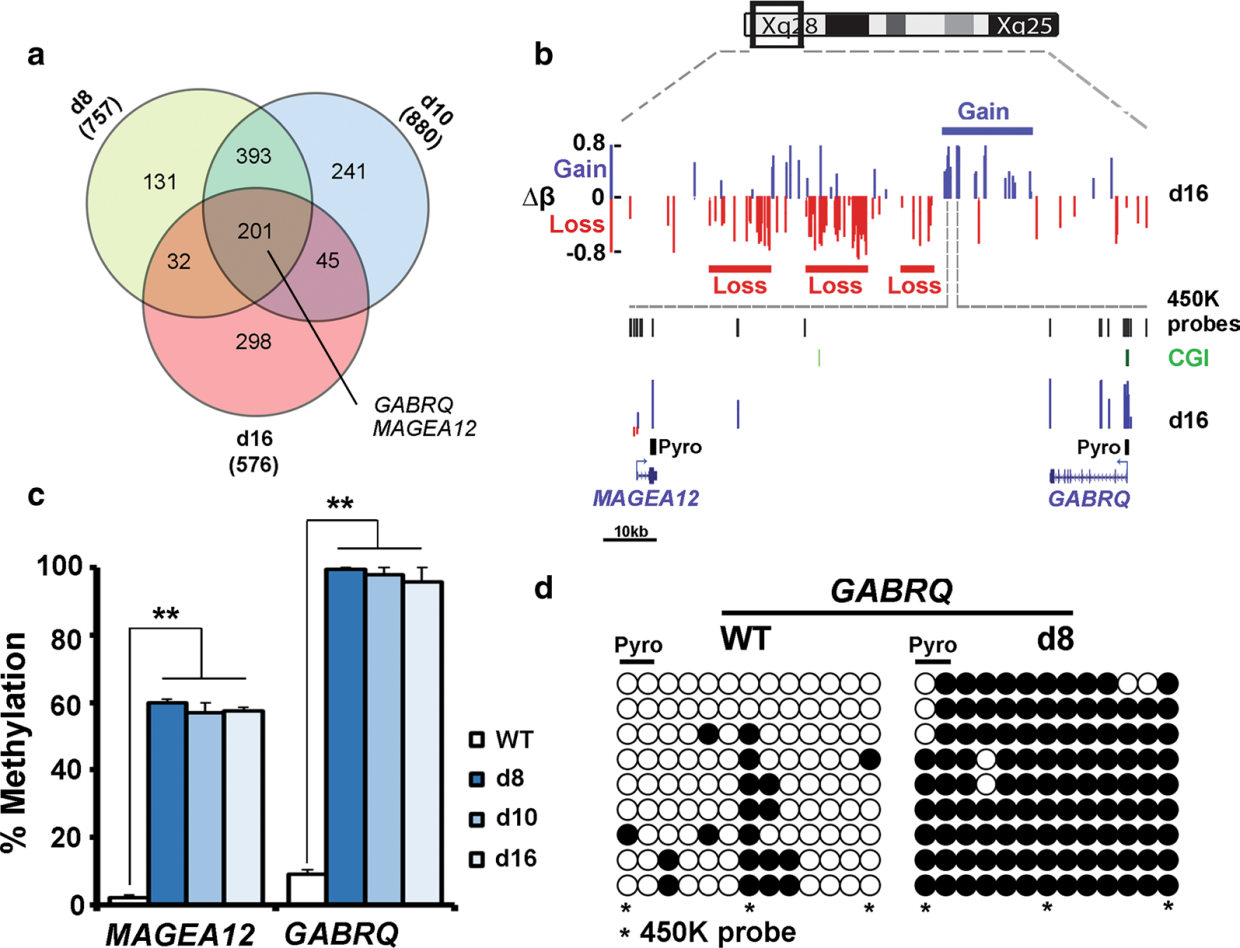
Gains in methylation on the X chromosome. **a** Sites reliably showing gain in methylation and located in promoters were analysed to identify those common to all three KD lines (*n* = 201). Some of these sites showing the greatest change in methylation were located on the X chromosome including *MAGEA12* and *GABRQ*. **b** Schematic showing the locations of the two genes adjacent to each other on X in a region showing gain in methylation. Tracks indicate the locations of all 450K probes and CGI; the positions of the pyroassays are also indicated; the scale bar pertains to the bottom part of the schematic; ∆*β*, change in beta value. **c** Methylation as determined by pyroassay at the two genes indicated in **a**, **b**. **d** Clonal analysis of *GABRQ* in WT and d8. Filled circles represent methylated sites, open circles unmethylated. The CpG which were also analysed by the pyroassay (pyro) and the 450K array (asterisk) are indicated

Given that there were considerable numbers of probes showing gain in methylation, but few of the GO classes from the RnBeads analysis contained testable targets by our criteria, we tried an alternative analysis as for the OR above. Sites associated with promoters and which showed reliable (FDR < 0.05) gains were identified in each KD line, and then the lists of cognate genes were compared to find those which were common to all three cell lines (Fig. 5a). Examination of the 201 promoters from this analysis (Additional file 5: Table S2) failed to show any significantly enriched terms in DAVID. However, several of the genes showing the greatest gain in methylation were located on the X chromosome, including *GABRQ* and members of the *MAGE* family of cancer/testis antigens such as *MAGEA12*. Mapping of FDR sites to the X chromosome showed that adjacent domains could vary in methylation level by more than 80% in either direction (Fig. 5b). Pyroassays for *GABRQ* and the neighbouring *MAGEA12* gene confirmed significant gains in methylation at the *GABRQ* promoter and in the *MAGEA12* gene body (Fig. 5c). Clonal analysis for *GABRQ* indicated a uniform increase in methylation (78 vs. 16%) across all adjacent CpG at this locus (Fig. 5d). Both direction and degree of change in methylation were highly correlated between pyrosequencing and the 450K array across all sites which were covered by both types of assay (*r *= 0.916 for loss of methylation *r *= 0.818 for gain in methylation).

### Transcriptional changes are enriched at cancer/testis antigen genes on X and Y

**Fig. 6:**
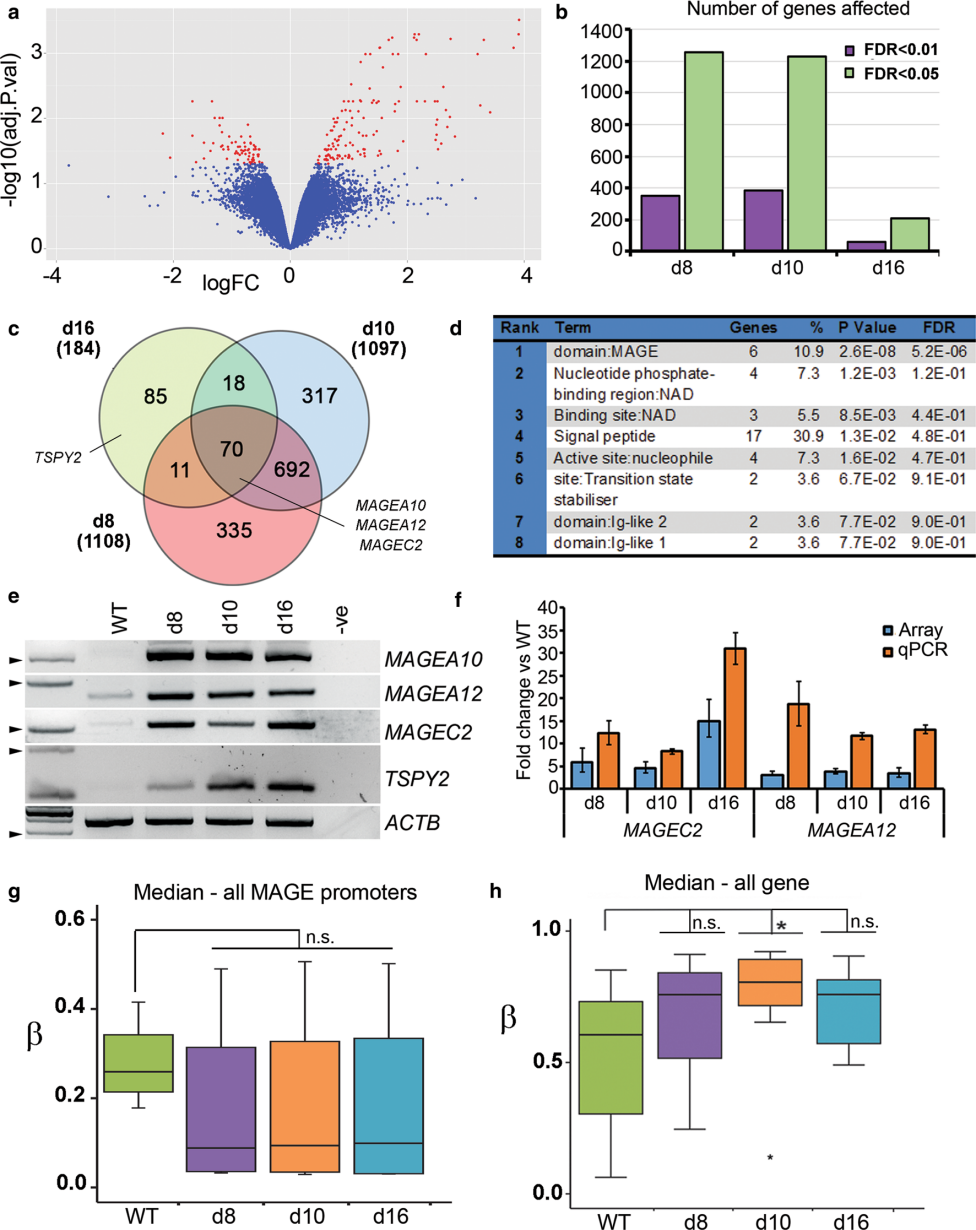
Transcriptional dysregulation of genes on the X and Y chromosomes correlates with methylation changes. **a** Volcano plot showing log fold change (FC) in transcription as measured by HT12 array versus FDR-corrected significance values: genes with > 2FC and FDR < 0.05 are highlighted in red. **b** Numbers of dysregulated genes at different FDR thresholds for the different KD lines. **c** Genes common to more than one KD line at FDR < 0.05; total numbers in each cell line are indicated in brackets. **d** Ontology enrichment output from DAVID for the genes common to all KD lines. **e** RT-PCR analysis of the three *MAGE* genes on X and a member of the *TSPY* gene family on Y highlighted in DAVID analysis (C). *ACTB* is a loading control; −ve, negative control lacking cDNA. A 100-bp ladder is shown at left with the 200-bp band indicated by an arrowhead. **f** Transcription levels of indicated MAGE genes from the HT12 array or by qPCR. Error bars are 95% CI for the array, SEM for qPCR; fold change was significant (*p* < 0.05) in all cases. **g** Median *β* values on 450K array for probes at MAGE promoters were decreased, though failed to reach significance. **h** Gene body methylation was increased in transcriptionally upregulated genes

**Fig. 7:**
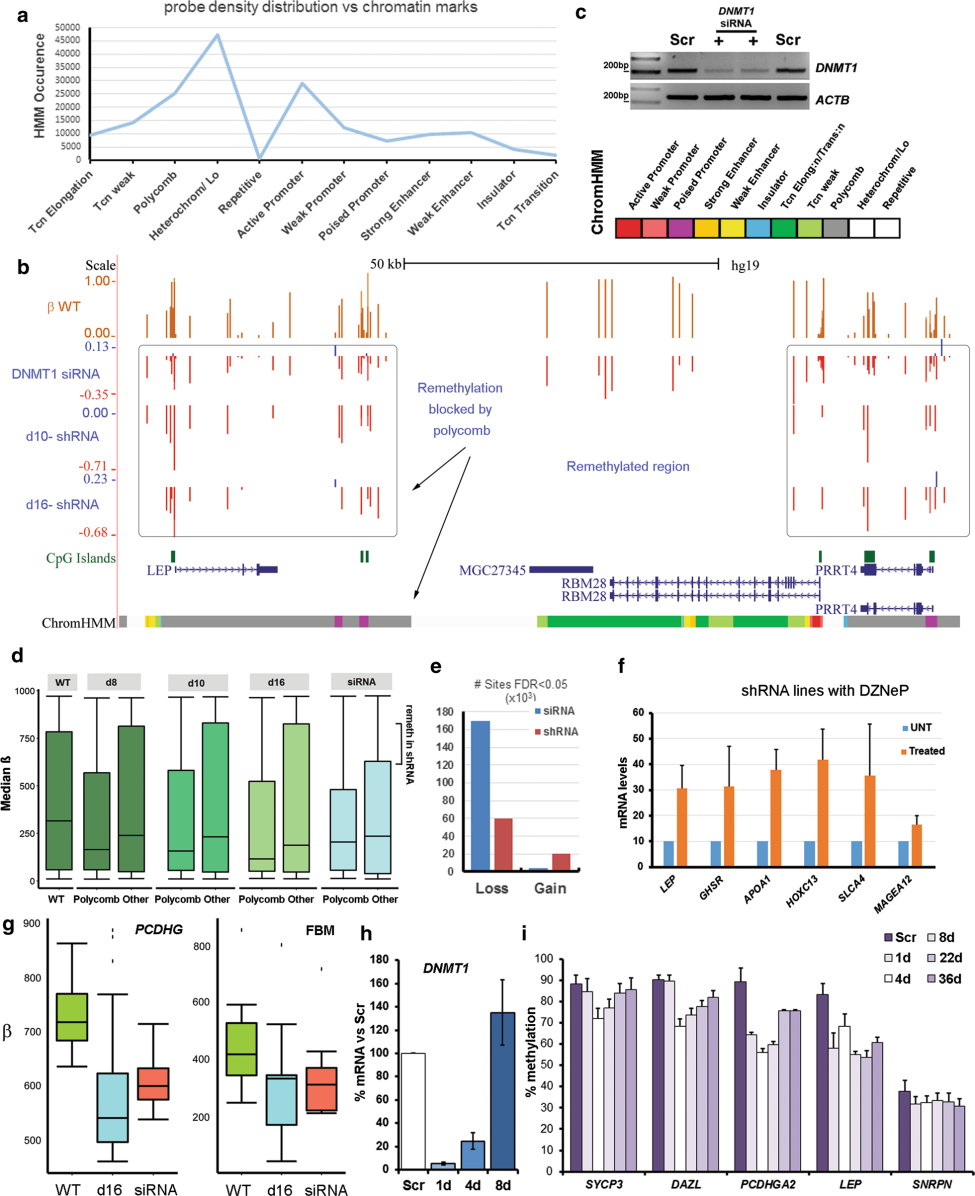
Methylation loss is concentrated at regions normally repressed by polycomb. **a** Distribution of probes showing significant loss per chromatin state—numbers of probes are shown at left, chromatin states below: tcn, transcription; heterochrom/Lo, heterochromatin or low signal; repetitive, repeat DNA. **b** Region around the *LEP* gene: tracks as before, with the addition of data from cells treated with siRNA for 72 h (top). A track showing ChromHMM chromatin states from NHLF foetal lung fibroblasts is shown at bottom: grey, polycomb-repressed; green, transcriptionally active (full colour key at top right). **c** DNMT1 mRNA levels by qPCR following treatment with siRNA (+) for 72 h compared with scrambled control (Scr). ACTB is shown as a control; ladder as above. **d** Median *β* values for all regions (WT) compared to medians for polycomb-repressed regions (Polycomb), or all other regions (Other) in the cell lines indicated at top; remeth, remethylated. **e** Numbers of probes showing loss and gain in methylation in hTERT cells following treatment with siRNA for 72 h compared with the shRNA lines (averaged); #, number. **f** mRNA levels for the indicated genes in shRNA lines treated with the EzH2 inhibitor DZNeP; UNT, untreated; bars represent SEM, experiment carried out in duplicate. **g** Median *β* values for all variable exons at the *PCDHG* locus (left) and for fat/body mass genes (FBM, right): compare d16 shRNA lines with cells treated with siRNA. **h** *DNMT1* mRNA levels in WT cells exposed to siRNA for 48 h, then allowed to recover in normal medium; comparisons were made to a scrambled siRNA negative control (Scr). **i** Methylation levels by pyroassay at the loci indicated during the transient KD and recovery shown in (**h**); timepoints are in days. All loci showed significant loss of methylation: *LEP* and *SNRPN* showed no significant gain versus lowest methylation level, while *PCDHGA2* showed no significant gain between d22 and d36

To see whether methylation changes were accompanied by large-scale changes in transcription, we carried out a genome-wide screen using the HT12 array which assays most RefSeq genes. Figure 7a shows the distribution of changes comparing d8 and WT: genes which showed > 2 fold change (FC) and with scores of *p* < 0.05 are highlighted, with the greater spread to the right indicating a greater tendency to derepression. Relatively small numbers of genes were affected (Fig. 6b), particularly at higher stringency (FDR < 0.01), and d16 showed fewest dysregulated genes. To determine common targets, we looked for shared genes (Fig. 6c). DAVID analysis on the genes common to all three (*n* = 70; Additional file 6: Table S3) indicated significant enrichment for genes coding for MAGE domains (Fig. 6d). MAGE genes on the X chromosome were previously identified as showing large changes in methylation (Fig. 5): also appearing here was a *TSPY* family member (Table 1, Fig. 3f). Upregulation of members of these gene classes could be verified by RT-PCR (Fig. 6e) and showed similar direction of change to the array, and greater magnitude, by RT-qPCR (Fig. 6f). Consistent with the transcriptional upregulation, median methylation levels at the promoters of these genes were lower than WT (Fig. 6g). Interestingly, there was an overall increase in intragenic (as opposed to promoter) methylation in the larger group of transcriptionally dysregulated genes common to d8 and d10 (*n *= 764, see Fig. 6h and Additional file 6: Table S3), which may reflect increasing gene body methylation accompanying transcription.

### Regions hypomethylated in shRNA lines correlate with polycomb repression

To investigate why losses in methylation occurred at the same positions in all KD lines, we used ENCODE data to look at chromosomal distribution, replication timing and chromatin features which might be important, since the DNMTs have no DNA sequence specificity themselves. Of these, the chromatin marks were most informative, in particular the ChromHMM dataset on lung fibroblasts which partitioned the genome into different types of chromatin based on a set of distinguishing histone marks and other features [43]. This indicated that probes significantly losing methylation in our shRNA lines are most densely distributed across regions which are normally polycomb-repressed or are heterochromatic/low-signal regions in lung fibroblasts (Fig. 7a). Specifically, many regions show a striking correlation between polycomb marking and methylation loss, such as the *LEP* and neighbouring *PRRT4* genes (Fig. 7b): in contrast, the intervening *MGC27345* and *RBM28* genes at that locus, which are highly methylated in WT cells (top track), show little or no loss of methylation and have chromatin marks associated with transcription.

These data suggested that polycomb-repressed regions might be more susceptible to demethylation than others. To test whether these regions lost methylation more readily than others, we treated hTERT1604 with siRNA for 72 h, which led to acute depletion of the *DNMT1* mRNA (Fig. 7c). We found, however, that there was little difference between polycomb-repressed and other regions in terms of demethylation in the siRNA-treated lines (Fig. 7d), in contrast to the shRNA lines where losses were concentrated at the former (Fig. 7d). This could also be seen at the *LEP* locus, where *MGC27345* and *RBM28* now showed loss of methylation following siRNA treatment (Fig. 7b, siRNA track). Also of note, almost no probes showed gains in methylation relative to WT in the siRNA cells (Fig. 7e), indicating that this effect is associated exclusively with chronic treatment. These results suggested that gains of methylation had occurred only in shRNA lines and had effectively restored methylation to near WT levels at most regions outside of those marked as polycomb-repressed.

Since transcriptional analysis did not highlight dysregulation of polycomb regions in shRNA cells (Fig. 6d), we tested to see whether polycomb-mediated repression was being maintained there in the absence of DNA methylation. To do this, we treated with DZNep, an inhibitor of EZH2, and confirmed the upregulation of a positive control gene *SLCA4* (Fig. 7f) as previously reported [44]. Likewise, *HOXC13*—a known polycomb target—showed derepression (Fig. 7f). The FBM genes marked by polycomb including *LEP* showed reactivation to a comparable degree to *SLCA4*, whereas the *MAGEA12* gene which is in a heterochromatic region not marked by polycomb showed little effect (Fig. 7f).

To further investigate the difference between acute and chronic DNMT1 depletion in these cells, we first examined the effects of acute depletion by siRNA on the loci identified in the stable lines: this confirmed that loci such as the clustered protocadherins and the fat/body mass genes also lose methylation on short-term depletion by siRNA (Fig. 7g). Following treatment, cells were then allowed to recover in the absence of siRNA for an extended period (36 days). DNMT1 levels returned to normal rapidly (Fig. 7h). Examination of the methylation response at various gene classes was very instructive. Germline genes (*SYCP3*, *DAZL*), which are known to become de novo methylated to high levels during somatic differentiation [5, 34], showed initial loss versus a scrambled control (Scr), followed by remethylation over time to near WT levels (Fig. 7i), confirming that the hTERT cells possess sufficient de novo activity to remethylate the genome, as already suggested (Fig. 7b–e). Imprinted genes are normally unable to regain methylation somatically [45], and we could confirm that the *SNRPN* imprint control region failed to remethylate (Fig. 7i). The polycomb-marked genes *LEP* and *PCDHGA2* were also refractory to de novo methylation, either showing no gain (*LEP*) or reaching a plateau at an intermediate level of recovery only (*PCDHGA2*) (Fig. 7i).

### Gain in methylation is associated with poised promoters in shRNA lines

**Fig. 8:**
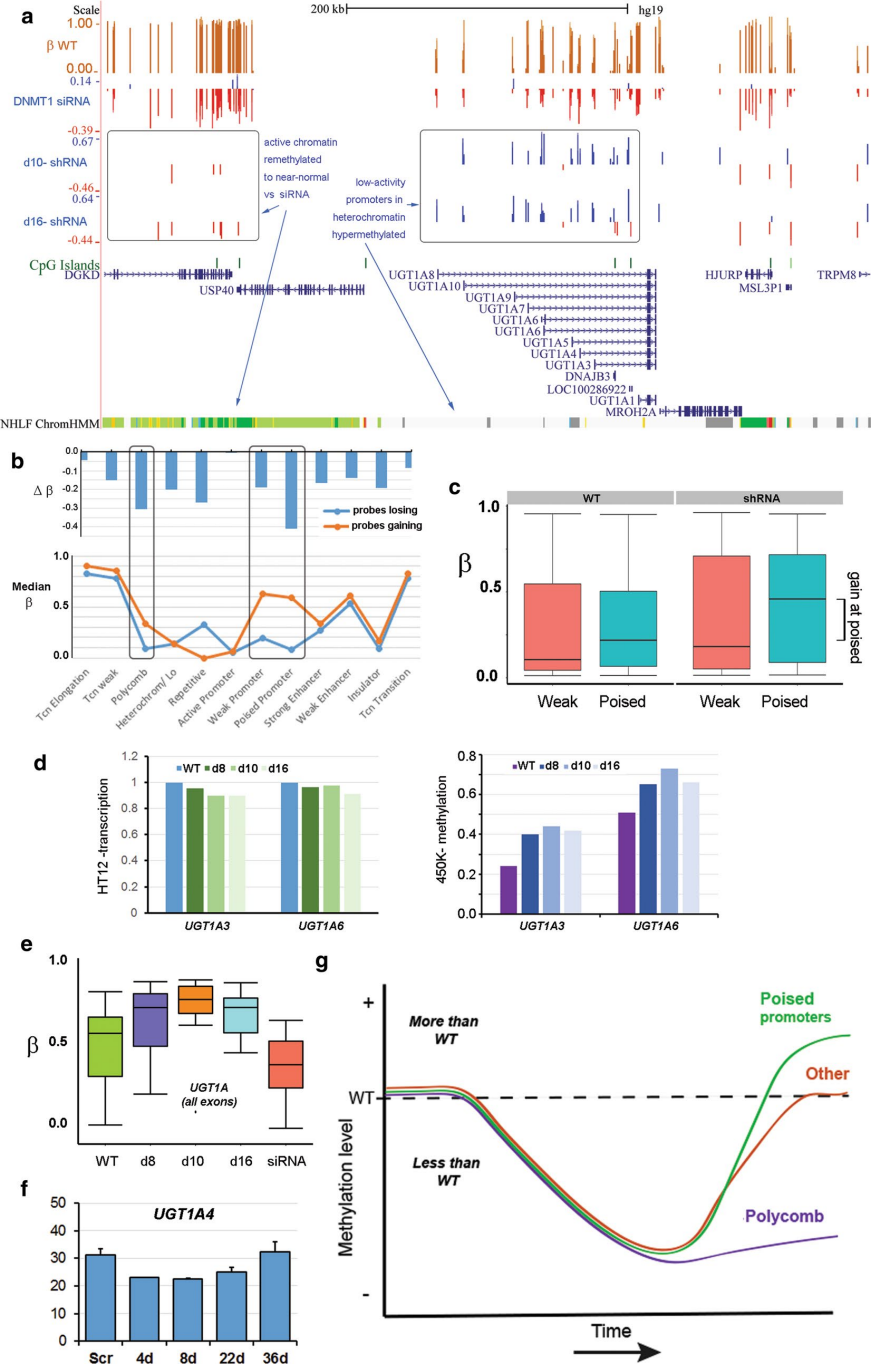
Methylation gain is concentrated at poised promoters. **a** *UGT1A* locus showing siRNA treatment data (top), shRNA lines (middle) and chromatin states (bottom); grey, heterochromatin/low signal; green, transcriptionally active (for full key see previous fig). **b** Median *β* levels for probes gaining and losing in shRNA lines (bottom) and median changes in methylation (∆*β*) versus WT for different chromatin states. **c** Boxplots of methylation values for probes falling within weak and poised promoter chromatin regions in WT or shRNA lines (averaged). **d** Transcription at the *UGT1A3* and *UGT1A6* genes decreases (relative to WT, set to 1) in all three shRNA lines as methylation (*β* value) increases, as indicated by HT12 and 450K arrays, respectively. **e** Median methylation (*β*) across all *UGT1A* exons decreases in siRNA-treated cells, but shows gains in all shRNA lines. **f** Methylation at *UGT1A2* during the transient KD and recovery experiment shown in Fig. 7h, i; differences are significant between control (Scr) and d4, but not Scr versus d36. **g** Model for methylation changes which occurred over time following chronic (shRNA) depletion of *DNMT1*: while polycomb-marked regions (purple) resisted remethylation, most regions (“other”, red) regained normal or near-normal levels, while poised promoters (green) tended to become hypermethylated

Having established that loss of methylation in shRNA lines is linked to polycomb repression, we wished to determine what features are associated with gains in methylation in these chronically depleted cell lines. As indicated, gains were not seen genome-wide following acute depletion using siRNA (Fig. 7e) and specific loci such as *UGT1A* showed instead loss of methylation on acute treatment (Fig. 8a, siRNA track), suggesting that hypermethylation is associated with longer-term culture of the shRNA-containing cell lines. To investigate what features might be associated with such loci, we looked to see which chromatin states in shRNA lines showed the highest median *β* for probes which gained methylation and the largest difference in methylation (Fig. 8b). This identified weak and poised promoter categories, and comparing shRNA lines to WT (Fig. 8c), the median values were more different for poised than for weak promoters (0.4 vs. 0.2, Cohen’s *D* test). These results suggested that poised promoters attract de novo methylation particularly strongly. Consistent with this, hypermethylation in the shRNA lines is centred around the *UGT1A* promoters and not the common 3′ exons (Fig. 8a). A heterochromatic location may contribute to over-methylation, since genes in adjacent active chromatin show restoration of normal methylation (Fig. 8a, compare siRNA to d10, d16 for *DGKD*), but not hypermethylation. While *UGT1A* transcription levels were very low compared to expressing cells by RT-qPCR (not shown), available HT12 array data showed a consistent decrease in transcription in all three shRNA lines (Fig. 8d, left), correlated with gains in methylation at the cognate promoters (Fig. 8d, right).

Further analysis confirmed that while gains in methylation were seen across all the UGT1A exons in all shRNA lines (Fig. 8e), all of these exons showed a loss of methylation following acute depletion with siRNA. We took advantage of our transient depletion and recovery experiment (Fig. 7h, i) to examine levels of methylation at *UGT1A4* using pyrosequencing: this showed that while the region indeed loses methylation on acute depletion, it undergoes steady de novo methylation following recovery and at day 36 was the only gene examined whose methylation exceeded that seen in the scrambled control (32.4 vs. 31.3%), suggesting that these genes are indeed susceptible to hypermethylation.

One possible reason for the gains in methylation seen in the shRNA lines could be over-expression of a de novo enzyme. Previous reports have indicated that between them, DNMT3B and DNMT1 account for the majority of methylation in cultured adult human cells and that there may be a role for DNMT3B in maintenance as well as de novo methylation [27]. We saw little change in *DNMT3B* levels in the *DNMT1* KD lines from the HT12 transcriptional array (Additional file 7: Fig. S4A) or RT-PCR (not shown), indicating that gains in methylation are not due to *DNMT3B* over-expression. To investigate a possible role in maintenance methylation, we carried out a transient siRNA treatment and could achieve robust knockdown of *DNMT3B* in the cells (Additional file 7: Fig. S4B). While some germline genes showed little effect, loci previously shown to require DNMT3B including *D4Z4* and *NBL2* did show loss of methylation (Additional file 7: Fig. S4C), confirming that we had achieved a functional depletion. Examination of the loci identified in our *DNMT1* shRNA clones showed that these loci also showed loss of methylation in *DNMT3B* KD cells (Additional file 7: Fig. S4C), suggesting that loci which remain hypomethylated in the shRNA clones also require input from DNMT3B to retain WT methylation levels.

## Discussion

### Summary and model

We and others have previously shown that acute depletion of DNMT1 using siRNA triggered the DNA damage response and cell cycle perturbations in human cell lines, making it difficult to identify genes which are directly controlled by methylation. Here we used isogenic shRNA-containing derivatives of a normosomic lung fibroblast cell line to look at the effects of chronic depletion of the protein. We characterised the alterations in methylation and transcription using microarrays in three different cell lines, processing them using a highly reproducible pipeline, and verified changes using locus-specific pyrosequencing or RT-qPCR assays. Additionally, we compared the effects on methylation of this chronic depletion to the effects of acute depletion using siRNA, as well as investigating possible contributions by DNMT3B. Finally, we investigated the correlations between chromatin state and DNA methylation and showed a role for polycomb-mediated repression at some of the loci.

Our results show that while both siRNA and shRNA-treated cells lose methylation overall as would be expected, only the latter show gains in methylation, most likely reflecting selection against the deleterious effects of hypomethylation during clonal expansion and culture. Figure 8e shows what we propose to have occurred: shRNA treatment gave initial widespread demethylation in all three clonal lines, since each line shows the presence of some highly demethylated sites distributed across the genome, but methylation seems to have recovered at most CpGs (Fig. 8e red line). Comparison to normal chromatin patterns in human lung fibroblasts indicated that remaining hypomethylation in the expanded cells was concentrated at regions normally marked for repression by polycomb (Fig. 8e purple line), while the smaller number of regions becoming hypermethylated relative to the parental cell line are associated with poised promoters (green line). TET expression was not detected, and the cells had little or no 5-hydroxymethylation (5hmC; data not shown), in keeping with other reports [46], suggesting that the hypermethylation does not represent 5hmC. Likewise, no over-expression of DNMT3B was detected.

In terms of what type of gene was particularly affected by chronic DNMT1 KD, the enrichment analyses and laboratory verification consistently pointed at the same small group of gene categories, namely (1)neuroepithelial genes, and in particular the protocadherins; (2) fat homoeostasis/body mass genes; (3) olfactory receptors; (4) the cancer/testis antigens; and (5) the *UGT1A* complex.

### Protocadherins are major targets of DNA methylation in human cells

Emerging evidence suggests that the clustered protocadherin genes may be central to specifying individual neural cell identity [47, 48] and they have been shown to become heavily methylated during embryonic development in mouse [49], suggesting that stable repression of non-transcribing copies is a programmed event during development. Recent work has shown that DNMT3B is important for de novo methylation at these loci and suggested that dysregulated expression may contribute to the phenotype in immunodeficiency, chromosome abnormalities and facial anomalies (ICF) syndrome [50], where *DNMT3B* is frequently mutated [51], and we found that depletion of DNMT3B was accompanied by loss of methylation at *PCDHGA2*. The *PCDHA* and *PCDHB* loci are heterochromatic and show persistent loss of methylation, as does the 5′ end of the *PCDHG* locus which is polycomb-repressed, but not the 3′ end which shows little loss of methylation and has instead chromatin marks associated with weak transcription (Additional file 3: Fig. S2B). Meehan and co-workers recently showed that long-term loss of DNA methylation in mouse *Dnmt1* −*/*− ES cells led to spreading of polycomb marks (in particular H3K27me3): their analyses singled out the *Pcdh* genes, which were heavily methylated in WT but not mutant ESC, as also shown by others [52]. Reddington et al. [17] also showed an increase in H3K27me3. A similar sequence of events in our human cells would cause an increase in H3K27me3 on *PCDH* genes and potentially help block remethylation. The sensitivity of the protocadherin cluster to methylation changes may explain why these genes are frequently identified in screens for differentially methylated loci in cancer [53]. The lack of derepression in our stable fibroblast cells is unsurprising here since expression of these genes is restricted to neurons [54]: they are also, with the exception of part of the *PCDHG* complex, heterochromatic rather than polycomb-repressed and may as such be harder to reactivate.

### Fat/body mass genes can be repressed by DNA methylation and polycomb

Currently, there is much interest in the possibility that altered diet, folate status or exposure to environmental toxins may lead to stable changes in the human methylome which particularly affect metabolic processes, as this offers an attractive mechanism by which it may be possible to partly explain the foetal origins of adult disease [55, 56]. Enrichment analysis in our cells identified the FBM genes involved in the common processes of lipid storage and body mass homoeostasis, including *LEP*, *GHSR* and the *APOC* cluster. These loci are readily demethylated on acute DNMT1 depletion and remain demethylated in chronically depleted cells where many other loci have recovered methylation. These loci are heavily marked by polycomb in normal fibroblasts, rather than being heterochromatic, which can potentially explain both their resistance to remethylation and their lack of transcriptional depression in the stable lines. In keeping with this, inhibition of the polycomb repressor EZH2 which generates H3K27me3 marks could reactivate these genes, as well as the canonical polycomb targets the *HOX* genes. These results suggest that in cells which have both DNA methylation and polycomb-mediated repression, both layers of repression must be removed to achieve gene activation. Interestingly a recent report by Hajkova and colleagues showed that reprogramming of germ cells in mouse also required both removal of DNA methylation and alteration of polycomb marks [57].

### Olfactory genes are methylated and largely inert

Olfactory receptors are also involved in specification of neural cell identity, where individual receptors are expressed in only a small group of cells in the olfactory epithelium [58]. They are largely monoallelically expressed, and methylation has been implicated as playing a role in their control [59, 60]. The OR gene family is the largest in the genome, with approx. 380 active members, many organised into “gene factories” where they are flanked by many more pseudogenes and repeats, such as the large cluster on chr11 [41]. These regions are often transcriptionally inert and heterochromatic, which together with the requirement for tissue-specific factors may explain their lack of derepression.

### Cancer/testis antigen genes are particular targets for demethylation and activation

The *TSPY* and *MAGE* genes fall into a functionally defined group known as the cancer/testis antigen (CTA) genes ([61, 62]; http://www.cta.lncc.br/) which are expressed during testis development normally, but which are aberrantly expressed in some tumours, such as melanoma and gonadoblastoma (e.g. *TSPY2*). This latter property makes them of particular interest for cancer immunotherapy, and monoclonal antibodies against some CTA members have already gained clinical approval [63]. CTA genes have been shown previously to lose methylation and become derepressed in several cancer cell types after treatment with the methyltransferase inhibitor 5′aza-2-deoxycytidine (Aza) [64–66] and in the HCT116 DNMT1 mutant line [66, 67] using locus-specific approaches. Our study (1) shows in an unbiased genomic screen that CTA genes are the genes most affected by loss of maintenance activity, (2) shows this for the first time in a normal, differentiated cell line and (3) highlights the subset of CTA genes which are particularly dependent on maintenance activity to keep them repressed. It is noteworthy that the majority of these genes are on the X chromosome, which shows major fluxes in methylation in our stable lines. The genes are largely associated with heterochromatin, rather than polycomb repression, and do not respond to EZH2 inhibition, but rather directly to loss of methylation, which may reflect some difference in heterochromatin marking on the X. Strategies to demethylate and turn on these genes in tumour cells (e.g. with Aza) to facilitate cancer vaccine development may be worthwhile to pursue, given that these genes are the most responsive to loss of methylation in our cell lines.

### UGT1A genes and other poised promoters are susceptible to hypermethylation

From the enrichment analysis, the *UGT1A* gene cluster was highlighted in terms of genes gaining methylation. These genes are known to be highly expressed in skin fibroblasts postnatally, and to be repressed in non-expressing tissues by methylation [68, 69]. The WT cells already had substantial levels of methylation but the increased methylation in the stable cell lines led to small but consistent decreases in transcription on the HT12 array, though levels were so low these could not be confirmed by Taqman qPCR (data not shown). It may be that the particular marks associated with a recent inactivation of the *UGT1A* cluster in the fibroblasts during adaptation to cell culture led to an increased de novo activity here, and in our transient KD experiment we saw the greatest gains in methylation at *UGT1A4*. Consistent with this, hypermethylation relative to the WT cells was associated with weak and poised promoters genome-wide, and the latter showed the greatest tendency to gain methylation above normal WT levels in the shRNA-containing lines.

### Lack of transcriptional changes in part due to polycomb

It is notable that while there was widespread changes in methylation in the KD cell lines, this was not accompanied by large-scale transcriptional derepression, with only a few hundred genes showing dysregulation, and the fold change in transcription being small. Of the four gene classes identified as most affected in terms of methylation, only one—that containing the *TSPY* and *MAGE* genes—showed robust transcriptional derepression. A lack of global changes in transcription, also reported by others [29, 70], is likely due to in part to the absence of transcription factors in fibroblasts needed to transcribe neural or adipocyte genes at high levels. However, many of the regions showing most persistent hypomethylation are polycomb-marked and this is likely to be sufficient in itself, as it is for example in *Drosophila*, to maintain repression of these genes. However, we could show that in the presence of an EZH2 inhibitor, polycomb-marked loci which lacked DNA methylation, such as those involved in fat homoeostasis/body mass regulation, became upregulated, along with canonical polycomb targets such as the HOX genes. Our results therefore indicate both that the polycomb system is sufficient in itself to repress and also that polycomb-repressed regions appear to be refractive to remethylation, which may be due to the action of FBXL10 [71]. It has previously been proposed that the two systems work in parallel, with their own sets of targets and a degree of mutual exclusivity [15–17]: our results would support such a conclusion.

### Comparison to other recent work

Two recent studies have also examined the effects of DNMT1 mutation on DNA methylation and gene transcription in human, albeit in cancer cells [29, 70]. Acute depletion of DNMT1 using an siRNA-mediated approach found, as we did, regions of low CpG density (open sea, etc.) to be most affected, but differed in finding more evidence for cell morphogenesis and phosphorylation pathways being affected [70]. This might reflect differences between acute and chronic depletion and the high levels of cell death during acute depletion. Blattler and colleagues [29] also found that relatively few genes were dysregulated in *DNMT1/3B* double KO HCT116 cells, but some cancer/testis genes (the related GAGE genes) were upregulated, along with Krüppel-associated box genes, while chaperonins figured prominently among down-regulated genes. The latter two gene classes may therefore be more dependent on DNMT3B, or the combination of DNMT1 and 3B, for their maintenance; alternatively the differences may be due to the experiment being carried out in colon cancer cells rather than, as here, in non-transformed fibroblasts.

## Conclusions

In conclusion, our study sheds new light on the loci which are most sensitive to sustained loss of maintenance activity in humans and shows an interplay between polycomb and DNA methylation-mediated repression in these differentiated cells.

## Additional files

**Additional file 1: Table S1.** Details of the primers used in this study.

**Additional file 2: Figure S1.** Variation between shRNA clonal lines. **(A)** Relative similarities between cell lines based on principal component analysis (PCA) of the 450K data; three independent cultures of each line were analysed. Note the clustering of lines d8R and d10R. The fraction of total variance explained by each component is indicated in brackets. (**B**) The 1000 sites most variably methylated between cell lines were used for hierarchical clustering. The location of sites with respect to CpG island is indicated at left. Beta values are depicted as shades from red (low) to blue (high).

**Additional file 3: Figure S2.** Changes in methylation levels by genomic element. **(A)** Protein levels in knockdown lines by western blotting. As a control HCT116 colon cancer cells which are WT or have a homozygous mutation in *DNMT1* (KO) are shown: the DNMT1-specific top band is indicated by the arrowhead at right. **(B)** Median levels of methylation are shown for each genomic element (listed at top). The positions of medians are also indicated at right (arrowheads). The differences between WT and KD medians were used to plot Fig. 1d. **(C)** Density distribution of methylation at the three main elements involved in gene regulation, shown by cell line. Demethylation seems most marked at gene bodies (Genes), indicated by increased density of probes at low methylation (β) values.

**Additional file 4: Figure S3.** Further analysis of enriched genes. **(A)**Total numbers of sites showing significant changes in methylation at different false discovery rates (FDR). Some sites showing gain were found in each KD cell line alongside the more numerous sites showing loss. **(B)** Differential methylation between WT and all KD lines using the 1000 best-ranking sites as identified by RnBeads (red). The majority of high-scoring sites common to all three lines lost methylation, but approx. one-third showed gain. **(C)** Methylation changes at neural identity genes on chromosome 5. Protocadherins in the α and γ families (*PCDHA* and* PCDHG* genes) have a clustered arrangement, while genes for the β family members are arranged individually. Tracks are as in Fig. 3. The position of the C class variable exons in the *PCDHA* and *PCDHG* clusters are also shown: gain in methylation relative to the siRNA-treated cells can be seen in the boxed regions, which includes the *PCDHG* constant exons, corresponding to transcriptionally active chromatin (green). **(D)** Median β values for gene bodies for olfactory receptors identified by DAVID: differences were significant by Mann-Whitney U (MWU). **(E)** Median β values for the promoters of genes in the histone modifier group identified by enrichment analysis in Table 1. No significant differences between WT and KD were found by MWU.

**Additional file 5: Table S2.** Details of the hypomethylated and hypermethylated genes from Figs. 3d and 5a, respectively.

**Additional file 6: Table S3.** Details of the genes showing transcriptional changes in KD cell lines from Fig. 6c.

**Additional file 7: Figure S4.** Role of DNMT3B in hTERT1604. **(A)** DNMT3B mRNA levels from the HT12 transcription array (3 probes) did not differ substantially in *DNMT1* shRNA cell lines from WT cells. **(B)** Successful depletion of *DNMT3B* mRNA using siRNA for 48hr, versus a scrambled control (Scr). **(C)** Methylation levels by pyroassay at the indicated loci: KD, knockdown. Methylation levels at 72hr were similar (not shown).
