# Supplementary figures and images for: Depletion of DNMT1 in differentiated human cells highlights key classes of sensitive genes and an interplay with polycomb repression
Source: Epigenetics Chromatin. 2018 Mar 29;11:12. doi: 10.1186/s13072-018-0182-4 (PMC5875016; doi:10.1186/s13072-018-0182-4)

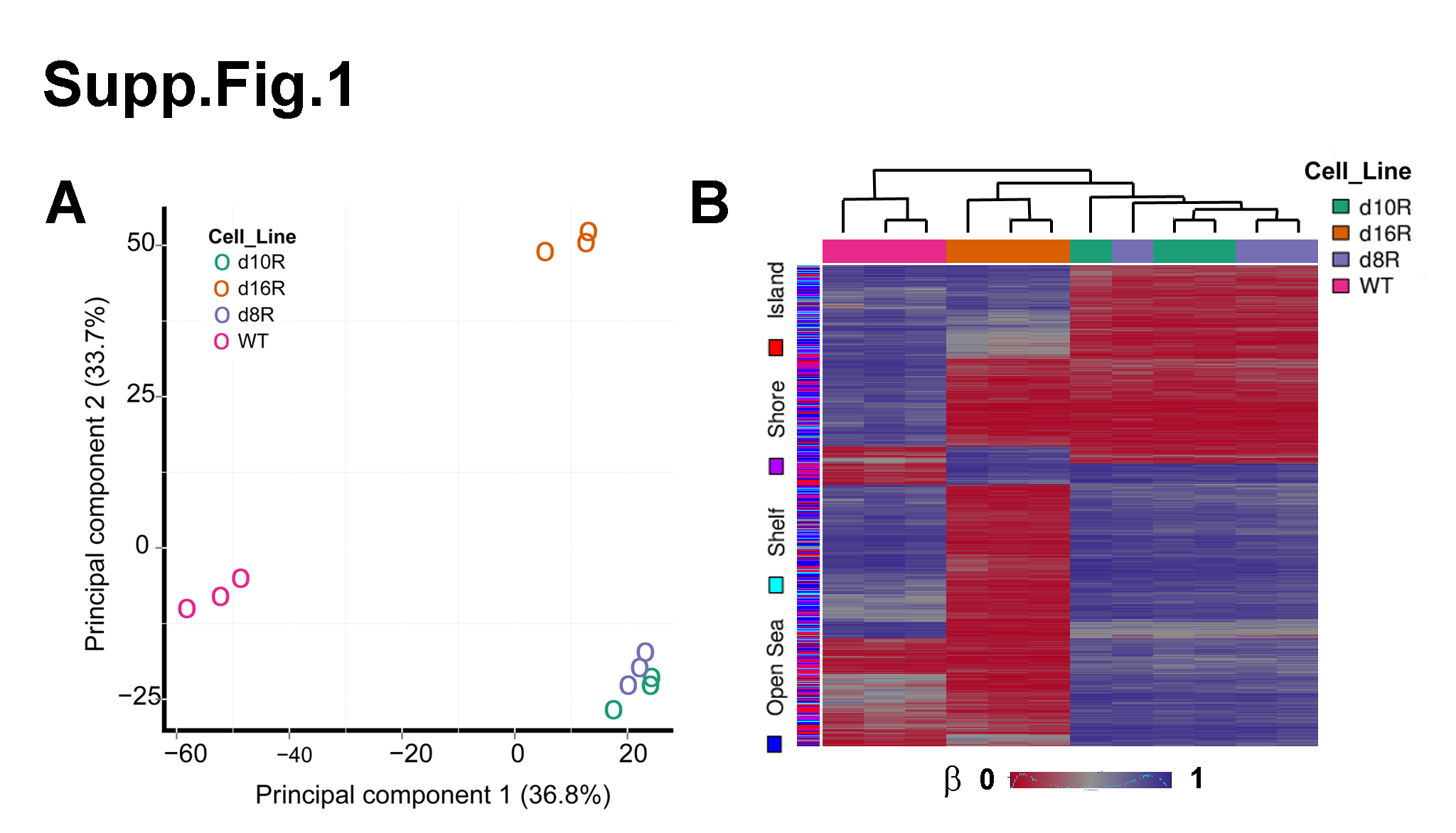

Supplement: Supplementary file 2 — Additional file 2: Figure S1. Variation between shRNA clonal lines. (A) Relative similarities between cell lines based on principal component analysis (PCA) of the 450K data; three independent cultures of each line were analysed. Note the clustering of lines d8R and d10R. The fraction of total variance explained by each component is indicated in brackets. (B) The 1000 sites most variably methylated between cell lines were used for hierarchical clustering. The location of sites with respect to CpG island is indicated at left. Beta values are depicted as shades from red (low) to blue (high). [file 13072_2018_182_MOESM2_ESM.tif]

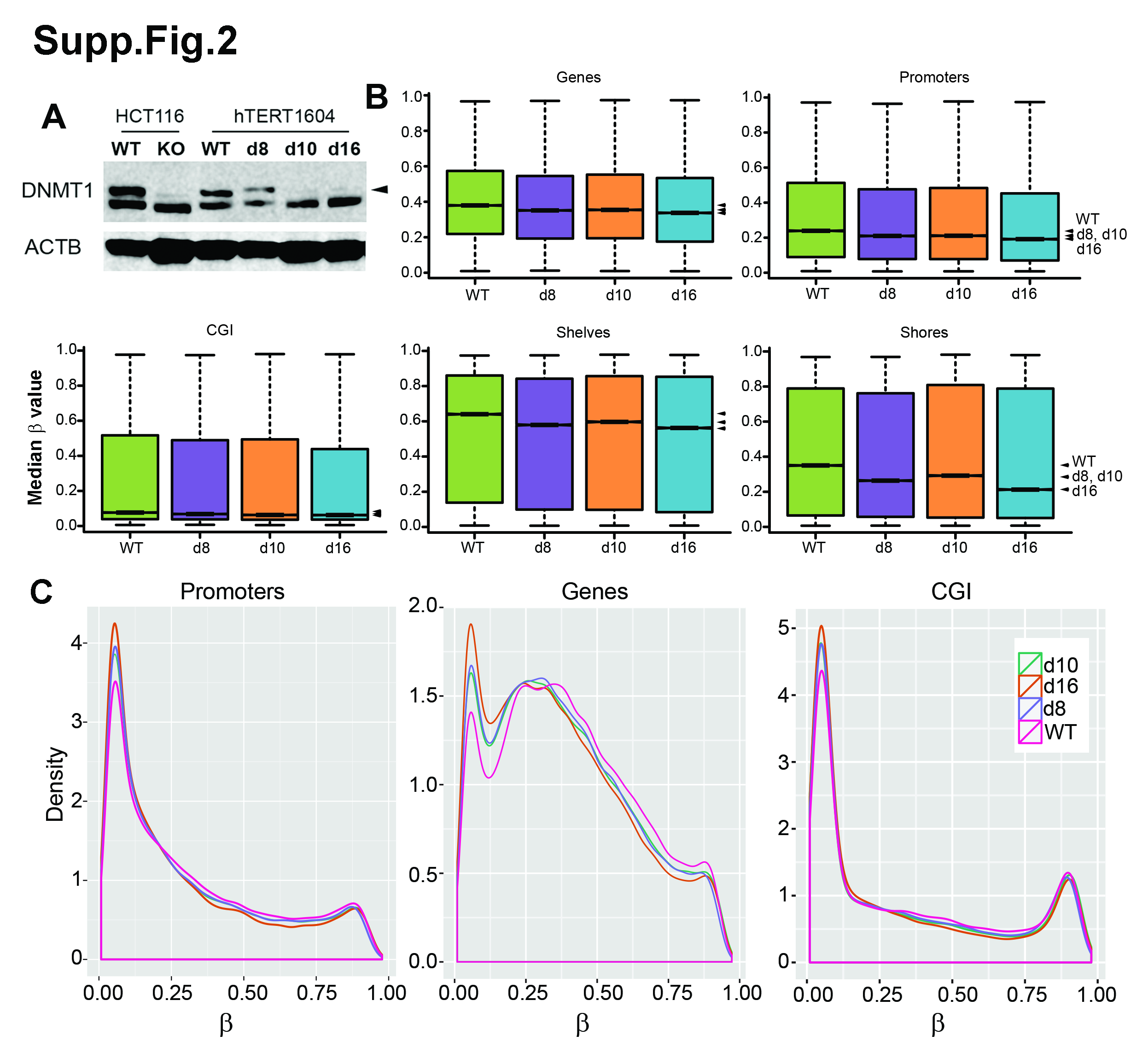

Supplement: Supplementary file 3 — Additional file 3: Figure S2. Changes in methylation levels by genomic element. (A) Protein levels in knockdown lines by western blotting. As a control HCT116 colon cancer cells which are WT or have a homozygous mutation in DNMT1 (KO) are shown: the DNMT1-specific top band is indicated by the arrowhead at right. (B) Median levels of methylation are shown for each genomic element (listed at top). The positions of medians are also indicated at right (arrowheads). The differences between WT and KD medians were used to plot Fig. 1d. (C) Density distribution of methylation at the three main elements involved in gene regulation, shown by cell line. Demethylation seems most marked at gene bodies (Genes), indicated by increased density of probes at low methylation (β) values. [file 13072_2018_182_MOESM3_ESM.tif]

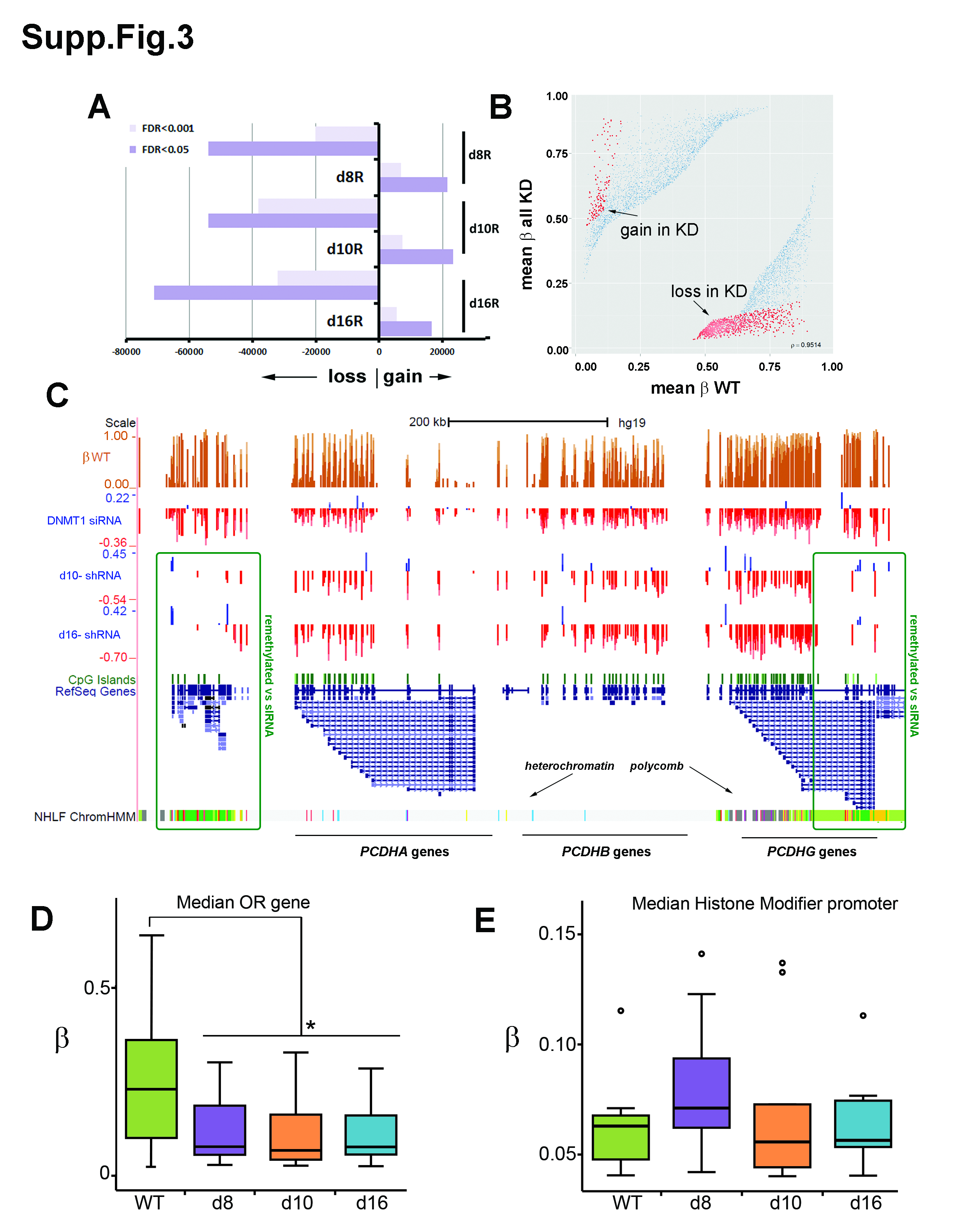

Supplement: Supplementary file 4 — Additional file 4: Figure S3. Further analysis of enriched genes. (A)Total numbers of sites showing significant changes in methylation at different false discovery rates (FDR). Some sites showing gain were found in each KD cell line alongside the more numerous sites showing loss. (B) Differential methylation between WT and all KD lines using the 1000 best-ranking sites as identified by RnBeads (red). The majority of high-scoring sites common to all three lines lost methylation, but approx. one-third showed gain. (C) Methylation changes at neural identity genes on chromosome 5. Protocadherins in the α and γ families (PCDHA and PCDHG genes) have a clustered arrangement, while genes for the β family members are arranged individually. Tracks are as in Fig. 3. The position of the C class variable exons in the PCDHA and PCDHG clusters are also shown: gain in methylation relative to the siRNA-treated cells can be seen in the boxed regions, which includes the PCDHG constant exons, corresponding to transcriptionally active chromatin (green). (D) Median β values for gene bodies for olfactory receptors identified by DAVID: differences were significant by Mann-Whitney U (MWU). (E) Median β values for the promoters of genes in the histone modifier group identified by enrichment analysis in Table 1. No significant differences between WT and KD were found by MWU. [file 13072_2018_182_MOESM4_ESM.tif]

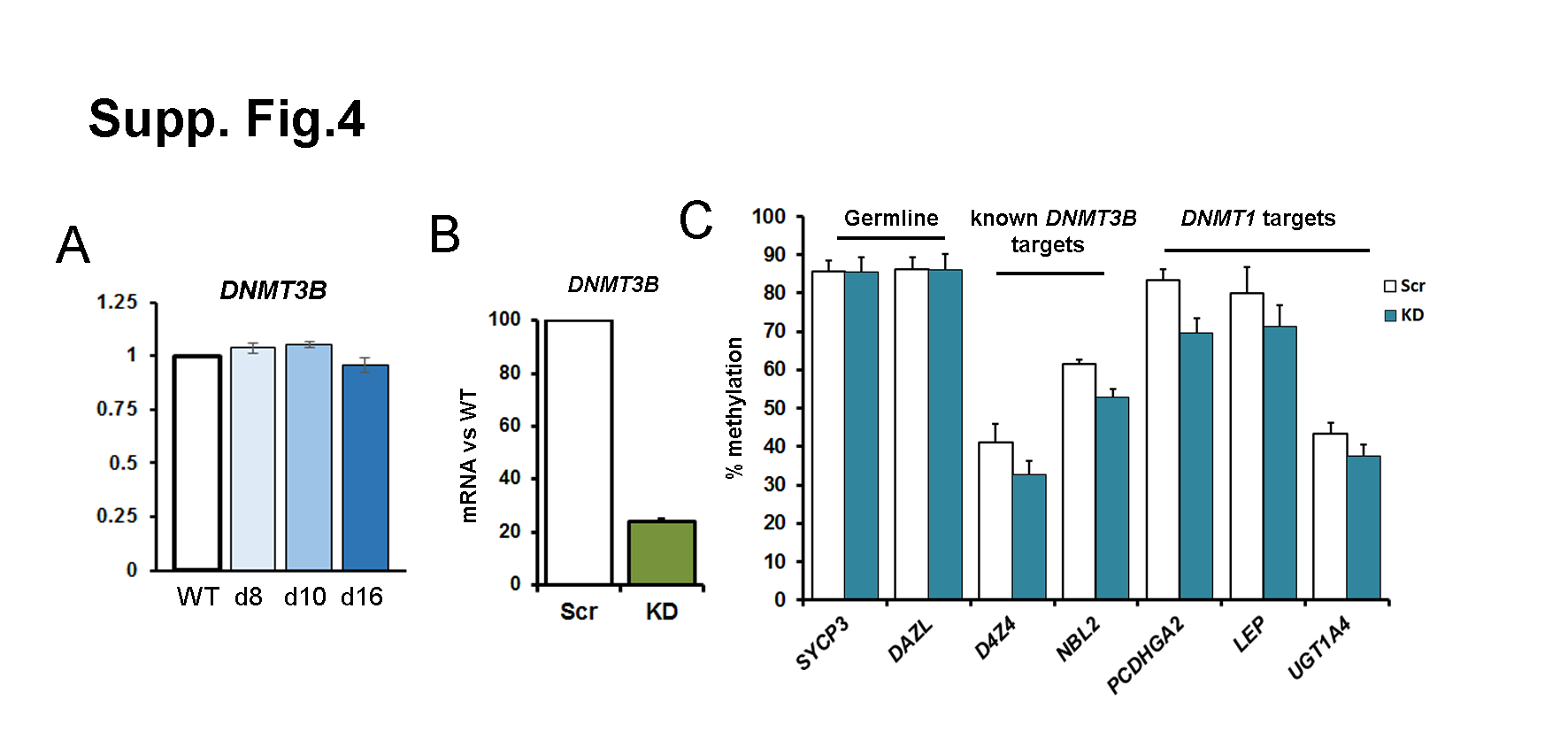

Supplement: Supplementary file 7 — Additional file 7: Figure S4. Role of DNMT3B in hTERT1604. (A) DNMT3B mRNA levels from the HT12 transcription array (3 probes) did not differ substantially in DNMT1 shRNA cell lines from WT cells. (B) Successful depletion of DNMT3B mRNA using siRNA for 48hr, versus a scrambled control (Scr). (C) Methylation levels by pyroassay at the indicated loci: KD, knockdown. Methylation levels at 72hr were similar (not shown). [file 13072_2018_182_MOESM7_ESM.tif]
